# Identification of a novel actin-dependent signal transducing module allows for the targeted degradation of GLI1

**DOI:** 10.1038/ncomms9023

**Published:** 2015-08-27

**Authors:** Philipp Schneider, Juan Miguel Bayo-Fina, Rajeev Singh, Pavan Kumar Dhanyamraju, Philipp Holz, Aninja Baier, Volker Fendrich, Annette Ramaswamy, Stefan Baumeister, Elisabeth D. Martinez, Matthias Lauth

**Affiliations:** 1 Department of Medicine, Philipps University, Institute of Molecular Biology and Tumor Research (IMT), Center for Tumor Biology and Immunology, Hans-Meerwein-Street 3, 35043 Marburg, Germany; 2 Department of Pharmacology, UT Southwestern Medical Center, 6000 Harry Hines boulevard, Dallas, Texas 75390-8593, USA; 3 Department of Surgery, Philipps University, Baldingerstraße 1, 35033 Marburg, Germany; 4 Department of Pathology, Philipps University, Baldingerstraße 1, 35033 Marburg, Germany; 5 Department of Biology, Philipps University, Karl-von-Frisch-Straße 8, 35043 Marburg, Germany

## Abstract

The Down syndrome-associated DYRK1A kinase has been reported as a stimulator of the developmentally important Hedgehog (Hh) pathway, but cells from Down syndrome patients paradoxically display reduced Hh signalling activity. Here we find that DYRK1A stimulates GLI transcription factor activity through phosphorylation of general nuclear localization clusters. In contrast, *in vivo* and *in vitro* experiments reveal that DYRK1A kinase can also function as an inhibitor of endogenous Hh signalling by negatively regulating ABLIM proteins, the actin cytoskeleton and the transcriptional co-activator MKL1 (MAL). As a final effector of the DYRK1A-ABLIM-actin-MKL1 sequence, we identify the MKL1 interactor Jumonji domain demethylase 1A (JMJD1A) as a novel Hh pathway component stabilizing the GLI1 protein in a demethylase-independent manner. Furthermore, a Jumonji-specific small-molecule antagonist represents a novel and powerful inhibitor of Hh signal transduction by inducing GLI1 protein degradation *in vitro* and *in vivo.*

## Abstract (web summary)

The regulation of the Hedgehog (Hh) pathway by the kinase DYRK1A is controversial. Here the authors uncover a novel inhibitory activity of DYRK1A that functions to destabilize the Hh downstream transcription factor GLI1 by destabilizing actin and preventing the nuclear accumulation of protective MKL1/JMJD1A complexes.

## Graphical abstract

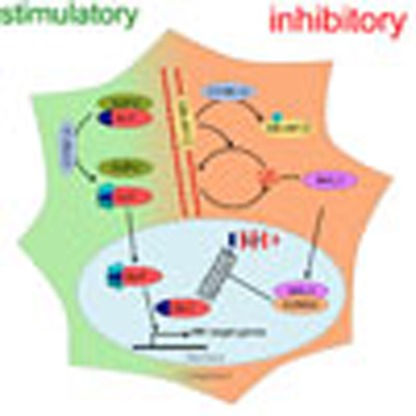

Down syndrome is caused by a trisomy of human chromosome 21, and is characterized by neurological, skeletal, cardiovascular and immunological defects^1^. In particular, the development of the brain and the cerebellum are impaired, resulting in microcephaly, central nervous system hypoplasia and mental retardation. The genomic region believed to be responsible for the Down phenotype is termed ‘Down syndrome critical region’ and it encompasses the dual-specificity-regulated kinase 1A (*DYRK1A*) gene locus^1^. The DYRK1A protein function is very sensitive to gene dosage and since Down syndrome patients have a higher DYRK1A expression due to the three *DYRK1A* copies in their genome, DYRK1A is considered constitutively overactive in their cells. As such, DYRK1A levels are thought to contribute to the overall Down syndrome phenotype, an assumption that is in agreement with *DYRK1A*-overexpressing transgenic mice showing deficits in memory and learning^2^,^3^. Furthermore, *DYRK1A* overexpression inhibits proliferation and correct differentiation of neural progenitor cells^4^,^5^,^6^,^7^.

The Hedgehog (Hh) signalling pathway is one of a few master regulators orchestrating major steps in vertebrate development, including the correct formation of the brain and the cerebellum^8^,^9^. The Hh pathway activity is dampened in cells derived from Down syndrome patients^10^ and in mouse models of this syndrome^11^. Importantly, certain morphological and cognitive deficits associated with Down spectrum could be ameliorated by the application of a synthetic Hh agonist^11^,^12^, suggesting a general Hh pathway suppression in these patients. This assumption is paradoxical as the DYRK1A kinase has been described as a stimulator of Hh pathway activity^13^,^14^, which is expected to lead to an increase in Hh signalling. Specifically, DYRK1A was shown to directly phosphorylate the Hh-regulated GLI transcription factors and promote their nuclear import^13^.

Hh signalling and its downstream GLI transcription factors are not only important for proper embryonic development, their overactivation has also been implicated in the formation of several tumour entities. Intriguingly, Down patients have a reduced risk for developing solid tumours, which is counterintuitive in light of the findings of DYRK1A promoting the activity of the oncogenic Hh pathway. Recent data actually propose *DYRK1A* to function as a tumour suppressor gene in medulloblastoma, melanoma, colon and pancreatic cancer^15^,^16^,^17^,^18^,^19^,^20^.

Given the discrepancy of data on the role of DYRK1A in the developmental and oncogenic Hh pathway, we set out to clarify this point. Our results reveal a dichotomous picture: on the one hand, DYRK1A is a stimulator of GLI1 activity. Specifically, DYRK1A promotes the nuclear translocation of the GLI1 transcription factor through phosphorylation of clusters of general nuclear localization signals located in the N terminus.

On the other hand, DYRK1A behaves as an inhibitor of Hh/GLI activity by acting negatively on the F-actin cytoskeleton. This effect involves ABLIM proteins, which we identified as novel DYRK1A phosphorylation targets capable of opposing the cytoskeletal effects of DYRK1A. Functionally, DYRK1A and ABLIM display competing effects on F-actin and on the nuclear translocation of the actin-sensitive transcriptional co-regulator MKL1 (MAL and MRTF-A). Nuclear MAL stimulates Hh/GLI activity in a serum response factor (SRF)-independent, but Jumonji domain-containing protein 1A (JMJD1A)-dependent manner. The histone demethylase JMJD1A directly binds to GLI1, protecting it from Itch/Numb-mediated proteasomal degradation, a process not requiring the enzymatic activity of the protein.

Thus, in terms of their impact on Hh signalling, our data suggest that DYRK1A should be considered mostly a negative regulator, whereas ABLIM proteins, MAL and JMJD1A, behave as positive Hh modulators. Interestingly, a small-molecule modulator of Jumonji enzymes acts as a potent Hh pathway inhibitor by inducing GLI1 protein degradation in cultured cells and *in vivo*.

These data provide a mechanistic framework on the negative regulation of Hh signalling by DYRK1A, explaining previous data derived from Down syndrome as well as cancer patients. Moreover, the findings can directly be exploited to establish Jumonji-binding molecules as novel and potent Hh pathway antagonists acting at the level of GLI transcription factors.

## Results

### DYRK1A phosphorylates nuclear localization clusters in GLI1

**Figure 1:**
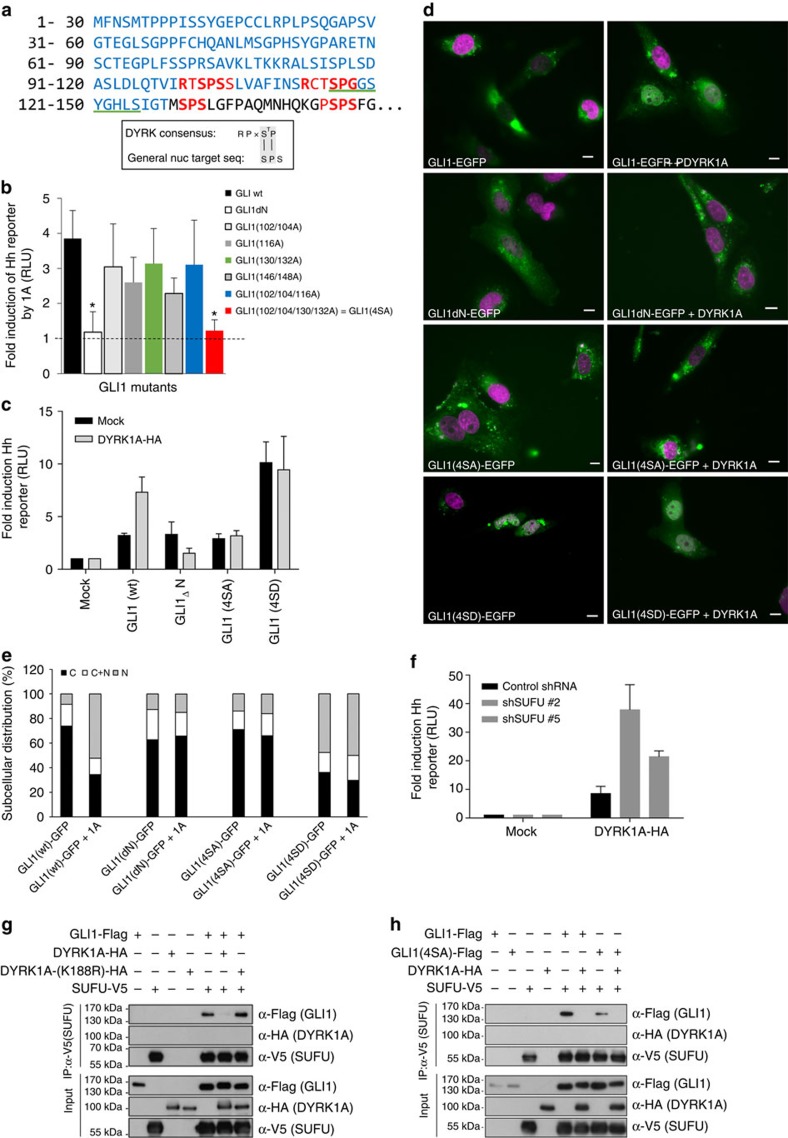
DYRK1A phosphorylates GLI N-terminal nuclear localization sequences. (**a**) Amino-acid sequence of the N-terminal domain of human GLI1. Red: potential general nuclear localization sequences. Green: SUFU-binding site. Blue: N-terminal region of GLI1. The box shows a comparison between the DYRK1A consensus sequence and the general nuclear targeting sequence. (**b**) Luciferase Hh reporter assay in NIH3T3 cells using the indicated Flag-tagged GLI1 mutants. Shown is the fold induction of luciferase acivity on DYRK1A co-transfection (mean of *n*=3±s.d.). (**c**) Hh reporter assay in NIH3T3 cells using the indicated GLI1-EGFP mutants. Shown is the reporter activity with or without DYRK1A co-expression (mean of *n*=3±s.d.). GLI1(4SA) and GLI1(4 s.d.) denote quadruple S102/104/130/132 mutations to Ala or Asp, respectively. (**d**) Fluorescence microscopy of U2OS cells transfected with the indicated GLI1-EGFP constructs (green) in the presence or absence of co-transfected DYRK1A. Nuclei appear in red/violet (NucRed647 stain). Scale bar, 10 μm. (**e**) Quantification of the microscopy results shown in **d**. Shown is the mean of two independent experiments with at least 50 cells counted in each experiment. (**f**) Hh reporter assay in Hek293T cells transfected with GLI1 plus the indicated shRNA constructs. Shown is the fold increase in luciferase activity on DYRK1A co-expression (mean of *n*=3±s.d.). Remark: due to the high reporter activity caused by *GLI1* overexpression, possible effects of *SUFU* knockdown on basal pathway activity could not be observed. (**g**) Co-immunoprecipitation between exogenous SUFU and GLI1 in HEK293T cells. The interaction is lost on DYRK1A co-expression, but not on kinase-dead DYRK1A^K188R^ co-transfection. Shown is a representative blot of three to four independent experiments performed. (**h**) Co-immunoprecipitation between exogenous SUFU and the GLI1(S102/104/130/132A) mutant in HEK293T cells. Shown is a representative blot of three to four independent experiments performed. *P<0.05; **P<0.005; ***P<0.0005 (student’s *t*-test).

Since DYRK1A had been described as a stimulator of GLI1 nuclear import^13^, we were interested in the identification of phosphorylation sites mediating this effect. Specifically, the N-terminal domain of GLI1 has been shown before to be crucial for DYRK1A-mediated stimulation^14^. Recently, general nuclear localization sequences (‘SPS’ motifs) were described which, when phosphorylated, promote the nuclear entry of ERK kinases^21^. Given the sequence similarity to the DYRK1A consensus sequence (RPxSP; refs 21, 22), we searched the N-terminal region of GLI1 for SPS (and SPS like) motifs. In fact, four SPS clusters (S102/104, S116, S130/132 and S146/148) could be found in the GLI1 sequence, some of them with a nearby arginine (which is part of the DYRK1A consensus sequence) and some of them close to the binding site of suppressor of fused (SUFU)^23^, a major negative regulator of Hh signalling^24^ (Fig. 1a). Sequential mutagenesis of the serine residues into non-phosphorylatable alanine revealed that no single SPS motif was responsible for DYRK1A-induced transcriptional stimulation. However, when both the S102/104 and S130/132 SPS clusters were mutated, DYRK1A was unable to enhance the transcriptional activity of GLI1 as measured in a luminometric Hh reporter assay (Fig. 1b). In line with an important role for these residues in mediating the DYRK1A effects, mutational introduction of phosphomimicking aspartates into these clusters generated a GLI1 protein with enhanced transcriptional activity that could not be further stimulated by DYRK1A (Fig. 1c). Moreover, fluorescence microscopy analysis of EGFP-tagged GLI1 mutants revealed that: (i) a S102/104/130/132A (S4A) mutant was not transported into the cell nucleus by DYRK1A and (ii) a S102/104/130/132D (S4D; phosphomimicking) mutant was constitutively localized to the nucleus even in the absence of DYRK1A (Fig. 1d,e). To prove a direct phosphorylation of the N-terminal domain of GLI1 by DYRK1A, we purified bacterially expressed protein and performed *in vitro* kinase assays together with recombinant DYRK1A followed by mass spectrometry. In addition, transfected HA-tagged full-length GLI1 protein (± co-transfected DYRK1A) was immunoprecipitated from cells and subjected to mass spectrometry. Both approaches revealed a single DYRK1A-mediated phosphorylation in the first (S102 in the S102/104 cluster) as well as within the second (S130/132) SPS cluster (although the identity of the exact phospho-residue in the latter cluster could not be resolved due to ionization difficulties while performing tandem mass spectrometry; Supplementary Fig. 1a,b). In summary, DYRK1A directly phosphorylates the GLI1 N-terminal domain on two SPS clusters leading to enhanced nuclear transport, increased activity of transfected GLI1 (Fig. 1b,c,d) as well as of endogenous signalling in NIH3T3 fibroblasts (Supplementary Fig. 1c).

The nuclear import of GLI1 as well as its transcriptional activity are strongly affected by the binding of SUFU^25^,^26^. Moreover, a SUFU-binding region is located between the S102/104 and S130/132 clusters identified here, motivating us to consider the effects of DYRK1A in the absence of SUFU. As can be seen in Fig. 1f, the DYRK1A-mediated stimulation of transfected GLI1 function is significantly increased when endogenous SUFU has been knocked down (for knockdown efficiency see Supplementary Fig. 1d). This prompted us to investigate whether GLI1 and SUFU might dissociate in the presence of DYRK1A. In line with this assumption, GLI1 and SUFU could readily be co-immunoprecipitated on co-transfection. In contrast, when DYRK1A was co-transfected, the GLI1–SUFU complex dissociated (Fig. 1g). The kinase activity of DYRK1A was required for this effect as co-transfection of a kinase-dead mutant (DYRK1A^K188R^; ref. 13) had no impact on the dissociation of the GLI1–SUFU complex. Next, we asked whether the kinase function of DYRK1A dissociates the GLI1–SUFU complex by direct phosphorylation of the GLI1 N-terminal SPS clusters. To this end, we repeated the co-immunoprecipitation experiment using the S102/104/130/132A mutant of GLI1. As can be seen in Fig. 1h, this mutant could still interact with transfected SUFU and this interaction could be broken up by co-transfected DYRK1A. Similar findings were made for the S4D-mutant of GLI1 (Supplementary Fig. 1e). Taken together, our data identify two SPS motif clusters in the N terminus of GLI1 as targets of DYRK1A phosphorylation, resulting in an increased nuclear import of GLI1. In addition, DYRK1A kinase activity promotes the dissociation of SUFU from GLI1, but this effect seems independent of the S102/104/130/132 clusters.

### DYRK1A can act to suppress endogenous Hh signalling

**Figure 2:**
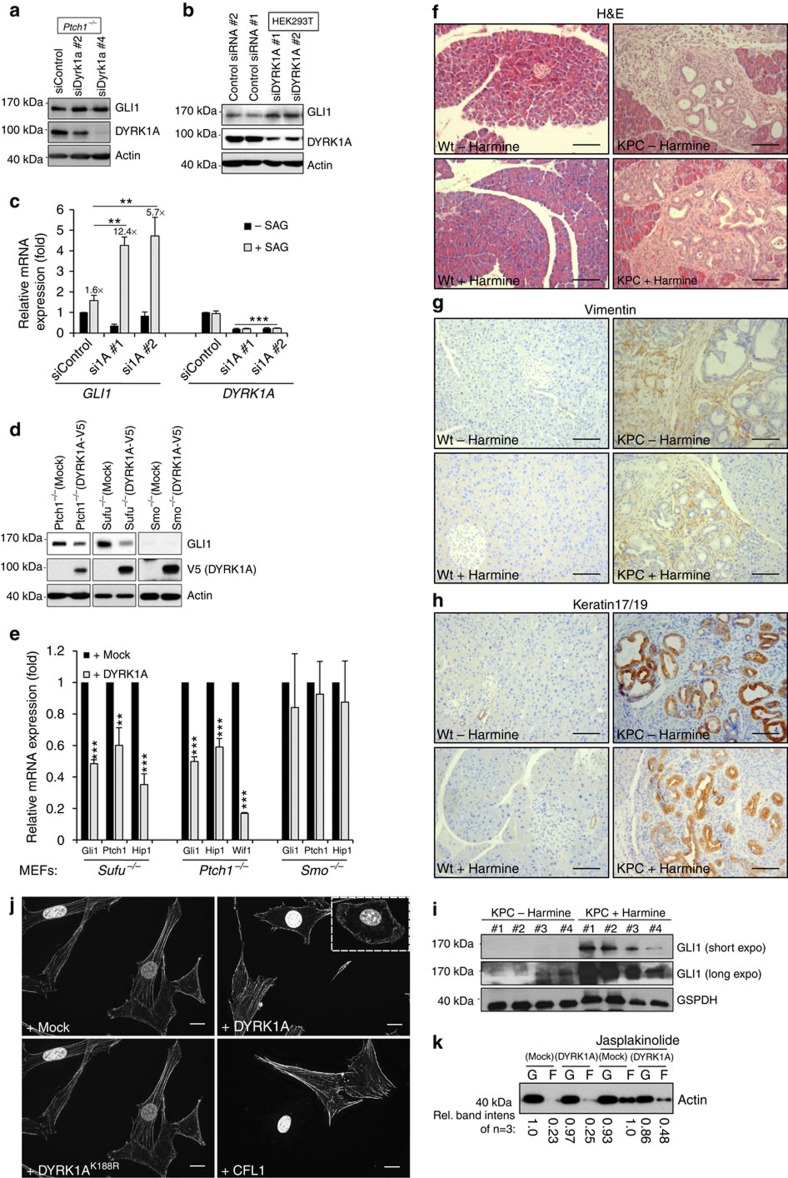
DYRK1A overexpression negatively affects the actin cytoskeleton. (**a**) Endogenous GLI1 and DYRK1A protein expression in siRNA-transfected *Ptch1−/−* MEF cells (1% FBS). (**b**) Endogenous GLI1 and DYRK1A protein expression in siRNA-transfected Hek293T cells. (**c**) *GLI1* and *DYRK1A* mRNA expression as determined by qPCR of siRNA-transfected DAOY cells. The fold induction on SAG of the Hh target gene *GLI1* is indicated above the bars (mean of *n*=3±s.d.). (**d**) Western blot of different MEF cell lines stably overexpressing V5-tagged DYRK1A (1% FBS). (**e**) Hh pathway target gene expression of the cells shown in **d** (mean of *n*=3–5±s.d.) (1% FBS). (**f**) H&E staining of mouse wild type (wt) or KPC pancreata from control or from harmine-treated animals. Scale bar, 100 μm. (**g**) Vimentin staining (brown) of mouse wild type (wt) or KPC pancreata from control or from harmine-treated animals. Scale bar, 100 μm. (**h**) Keratin 17/19 staining (brown) of mouse wild type (wt) or KPC pancreata from control or from harmine-treated animals. Scale bar, 100 μm. (**i**) Pancreatic GLI1 protein expression in KPC control and in harmine-treated KPC mice. (**j**) Three-dimensional deconvolution images of F-actin fibres (phalloidin) in NIH3T3 cells transiently transfected with the indicated constructs. Transfected cells were identified through co-transfection of a small amount of nuclear histone H2B–GFP plasmid. The F-actin severing Cofilin (CFL1) was transfected as a positive control. The inset in the upper right panel depicts a cell with clearly visible fragmented F-actin and a lack of stress fibres. Scale bar, 10 μm. (**k**) Cellular actin fractionation assay in NIH3T3 stably expressing empty vector control (mock) or DYRK1A. G, G-actin fraction; F, F-actin fraction. Jasplakinolide: 10 nM for 30 min. Shown is a representative anti β-actin western blot of three independent experiments; the mean value of the western blot band intensities of these three experiments is shown below. *P<0.05; **P<0.005; ***P<0.0005 (Student’s *t-*test).

We went on to verify the DYRK1A-induced stimulation of GLI1 in various knockout mouse embryonic fibroblast (MEF) cell lines and could find the expected increase in transfected GLI1 activity in *Ptch1−/−* and *Smo−/−* cells, but surprisingly not in *Sufu−/−* MEF cells, suggesting that the dissociation from SUFU might play a functional role in this process (Supplementary Fig. 2a,b). Since all our previous experiments were performed with transient transfection approaches, typically yielding very high expression levels, we were interested to assess the impact of endogenous DYRK1A on endogenous Hh signalling. We therefore knocked down *Dyrk1a* messenger RNA (mRNA) expression by two independent short interfering RNA (siRNA) constructs and measured the protein levels of GLI1, the best established Hh target gene. As cellular system we used cells with ligand-independent Hh pathway activation (*Ptch1−/−* and *Sufu−/−* MEF cells)^24^ and human Hek293T cells. To our surprise, knockdown of *Dyrk1a* did not result in a reduction of Hh pathway activity, but had either no effect or led to an increase in GLI1 expression (Fig 2a,b; Supplementary Fig. 2cd). We further verified this finding in human medulloblastoma cells (Daoy), in which the knockdown of *DYRK1A* led to an increased sensitivity towards the smoothened agonist (SAG) (Fig. 2c). To investigate the negative effects of DYRK1A on endogenous Hh signalling further, we generated *Ptch1−/−* and *Sufu−/−* MEF cells stably overexpressing moderate levels of DYRK1A. Interestingly, ectopic *DYRK1A* expression resulted in a suppression of endogenous Hh signalling in these cells, both on the protein (Fig. 2d) as well as on the mRNA level (Fig. 2e). Importantly, ectopic DYRK1A expression did not affect Hh target gene expression in *Smo−/−* cells, arguing that its effects are not mediated through canonical, SMO-dependent Hh signalling (Fig. 2e).

Thus, the net effects of DYRK1A on the Hh pathway might either be cell-type specific or be related to the expression levels of DYRK1A and/or GLI1. However, since many cell lines investigated by us showed evidence of a negative pathway regulation by DYRK1A, we speculated that this type of regulation might actually represent the majority of cases.

In an attempt to elucidate whether DYRK1A would negatively affect Hh signalling also under *in vivo* conditions, we made use of the LSL-Kras^G12D^; LSL-Trp^R172H^; Pdx1-Cre (KPC) mouse model^27^. We considered this model to be well-suited for our purpose as these animals develop pancreatic tumours in which epithelial cells secrete Hh ligands and the high number of surrounding stromal fibroblasts respond to these ligands^28^,^29^,^30^. We treated these mice (and control animals) with the DYRK1A-selective inhibitor harmine^22^,^31^ for 2 months and performed histology and GLI1 protein analysis. In line with recently published data on Hh signalling in pancreatic cancer^32^, we could not detect clear changes in the gross histology, in the degree of fibrosis, in disease staging or in the level of tissue inflammation on DYRK1A inhibition (Fig. 2f,g,h; Supplementary Fig. 2e,f,g). However, the amount of GLI1 protein was clearly increased in the harmine-receiving animals (Fig. 2i), suggesting a negative impact of DYRK1A on Hh signalling *in vivo*. In line with these findings of a negative Hh pathway regulation through DYRK1A, analysis of post-mortem brain samples from Down syndrome patients revealed a modest but significant reduction in expression of the Hh target genes *GLI1* and *PTCH2* in comparison with the normal brain^33^ (Supplementary Fig. 2h).

Next, we were interested to learn about the mechanism which DYRK1A uses to regulate Hh signalling. Since this kinase had been reported to affect the actin cytoskeleton in human and *Drosophila* cells^34^,^35^, potentially impacting on the Hh pathway, we wondered whether we can observe a similar effect. Indeed, transfection of NIH3T3 cells with a DYRK1A expression plasmid resulted in a striking rearrangement of the F-actin network with less F-actin fibres visible. This effect was dependent on DYRK1A’s kinase activity as transfection of a kinase-dead variant had no effect on the F-actin cytoskeleton (Fig. 2j; Supplementary Fig. 2i). This finding could also be quantified in independent assays in which F-actin and G-actin were separated by biochemical fractionation (Fig. 2k). In these assays, stabilization of F-actin bundles by application of Jasplakinolide was significantly less potent in cells with DYRK1A expression, arguing for an actin-destabilizing effect of this kinase.

### DYRK1A phosphorylates actin-stabilizing ABLIM proteins

**Figure 3:**
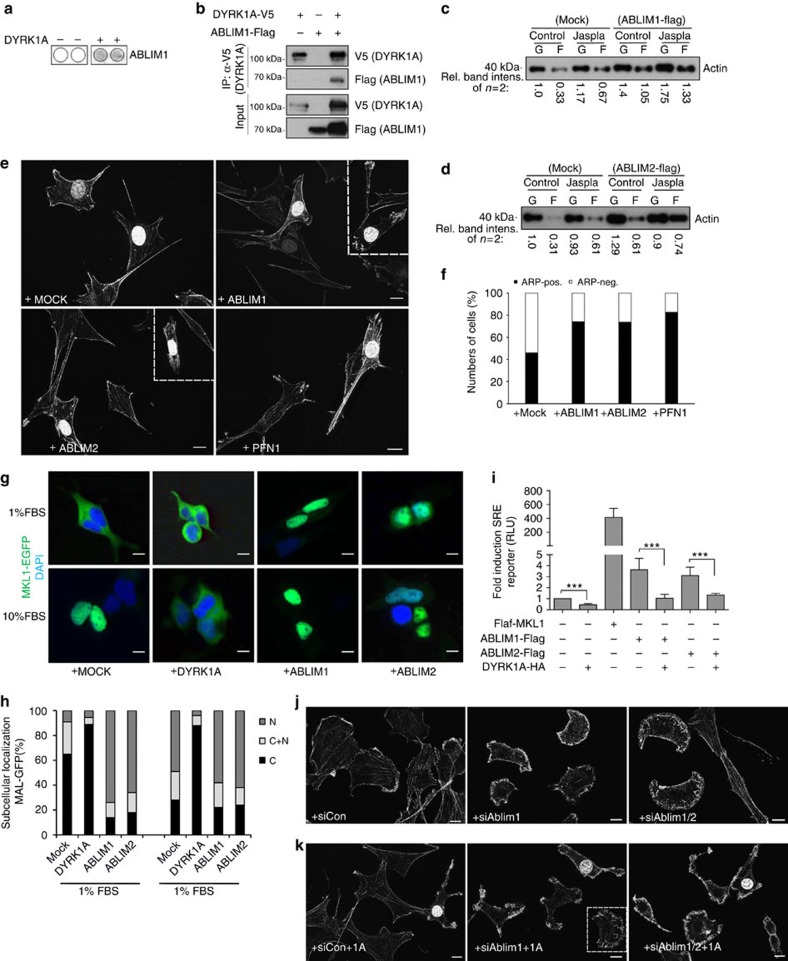
ABLIM proteins are DYRK1A substrates stabilizing F-actin. (**a**) Autoradiograph of protein microarray showing duplicate spots of recombinant ABLIM1 without (left) or with (right) recombinant DYRK1A, both with radioactive ATP. (**b**) Co-immunoprecipitation of exogenous DYRK1A and ABLIM1 proteins in Hek293T cells. Shown is a representative blot of three independent experiments. (**c**) Cellular actin fractionation assay in NIH3T3 stably expressing empty vector control (mock) or ABLIM1. Jaspla, Jasplakinolide 10 nM for 30 min. Shown is a representative β-actin western blot of two independent experiments (the mean band intensity is given below). (**d**) Cellular actin fractionation assay in NIH3T3 stably expressing empty vector control (mock) or ABLIM2. Jaspla, Jasplakinolide 10 nM for 30 min. Shown is a representative β-actin western blot of two independent experiments (the mean band intensity is given below). (**e**) Deconvolution images of F-actin fibres (phalloidin) in NIH3T3 cells transiently expressing the indicated constructs. Transfected cells were identified through co-transfection of nuclear H2B–GFP. The F-actin promoting Profilin (PFN1) was included as a positive control. Scale bar, 10 μm. (**f**) Quantification of the microscopy experiment depicted in **e** (mean of two independent experiments with at least 50 cells counted in each experiment). ARR, actin-rich region (lamellipodia, stress fibres, membrane ruffles or pronounced phalloidin positivity). (**g**) Subcellular localization of MKL1-GFP (green) in transiently transfected Hek293A cells. Representative pictures are shown. Nuclei appear in blue (4,6-diamidino-2-phenylindole). Scale bar, 10 μm. (**h**) Quantification of the experiment depicted in **f**. C, cytoplasmic; N, nuclear; N+C, nuclear and cytoplasmic localization of MKL1-GFP (mean of *n*=3 independent experiments. At least 100 cells were counted). (**i**) SRE (serum response element) luciferase reporter assay in Hek293T cells transiently transfected with the indicated constructs (mean of *n*=6±s.d.). (**j**) Phalloidin staining of siRNA-transfected NIH3T3 cells. Shown are representative images of two independent experiments. siABlim1/2: double knockdown of *Ablim1* plus *Ablim2*. Scale bar, 10 μm. (**k**) Phalloidin staining of siRNA/plasmid double-transfected NIH3T3 cells. The inset in the middle panel should help portrait the different phenotypes observed upon *Ablim1* knockdown. siABlim1/2: double knockdown of *Ablim1* plus *Ablim2.* Scale bar, 10 μm. *P<0.05; **P<0.005; ***P<0.0005 (Student’s *t*-test).

To get a clearer picture of the underlying mechanism of DYRK1A-related Hh effects, we further investigated its impact on the actin cytoskeleton. We aimed to identify possible DYRK1A protein substrates that may affect actin dynamics, and to this end we performed an *in vitro* protein microarray experiment with recombinant enzymatically active DYRK1A and ∼9,500 spotted protein substrates. Incubation with radioactively labelled ATP revealed numerous DYRK1A substrates including several splicing factors (as was already published; ref. 36), thus validating our screen (Supplementary Table 1). Intriguingly, actin-binding LIM protein 1 (ABLIM1), an actin-regulating protein^37^, was identified as a novel protein substrate of DYRK1A (Fig. 3a). Supporting a direct interaction of these two proteins, we could co-immunoprecipitate DYRK1A and ABLIM1 from cellular lysates (Fig. 3b). Interestingly, the expression of ABLIM1 led to an increase in cellular F-actin (Fig. 3c), contrary to the observations made with DYRK1A expression (Fig. 2g). Identical findings were made on expression of ABLIM2, a related family member (Fig. 3d). Staining for the F-actin network using fluorescently labelled phalloidin revealed that, when compared with mock-transfected cells, ABLIM-expressing cells showed more F-actin-rich structures such as membrane ruffles, stress fibres or lammellipodia (Fig. 3e,f).

The fact that DYRK1A reduced F-actin fibres and that ABLIM proteins increased F-actin formation suggested that the two proteins have opposing roles in cytoskeletal organization. To investigate this hypothesis further, we tested the effects of DYRK1A/ABLIM on the localization of the actin-regulated transcriptional regulator MKL1 (MAL and MRTF-A). MKL1 resides in the cytoplasm if cells are cultured under low serum conditions and it enters the nucleus when high serum activates Rho-dependent actin polymerization and F-actin formation^38^. As can be seen in Fig. 3g,h, DYRK1A expression blocked the serum/F-actin-triggered nuclear entry of green fluorescent protein (GFP)-tagged MKL1, in line with its negative role on the F-actin network. In contrast, the F-actin promoting ABLIM proteins favoured the nuclear import of MKL1, irrespective of the serum concentrations (Fig. 3g,h). These observations could also be verified in a functional luciferase assay measuring the activity of the SRF, the best characterized interactor of MKL1 (ref. 38): in line with our previous results, SRF/MKL1 activity was reduced on DYRK1A expression, whereas SRF/MKL1 activity was enhanced after ABLIM expression (Fig. 3i). Further supporting the opposing roles of DYRK1A and ABLIM, the ABLIM-mediated SRF stimulation could be antagonized by co-transfection of DYRK1A (Fig. 3i). An epistatic relationship between DYRK1A and ABLIM could also be found in RNA interference (RNAi) experiments: the knockdown of endogenous *Ablim1/2* reduced the number of stress fibres in fibroblasts (Fig. 3j; Supplementary Fig. 3a,b) and this phenotype could not be exacerbated by the concomitant transfection of DYRK1A expression plasmid (Fig. 3k; Supplementary Fig. 3b). Extending our data on DYRK1A, actin and MAL/SRF, publicly available array data of human Down syndrome patient brain tissue demonstrate a reduced expression of numerous MKL1/SRF target genes (Supplementary Fig. 3c).

Given that DYRK1A regulates Hh signalling, the prediction from the data presented here would be that ABLIM and other actin-regulating proteins represent novel Hh regulators. In fact, we found that actin-regulating proteins (DYRK1A, ABLIM1, NWASP and PFN1) as well as an actin-modulating drug (Latrunculin B) can indeed modulate endogenous Hh signalling (Supplementary Fig. 3de,f). In summary, our data suggest a model in which DYRK1A regulates the actin cytoskeleton, at least in part, through phosphorylating and functionally inactivating ABLIM proteins.

### MKL1 and ABLIM are positive regulators of Hh signalling

**Figure 4:**
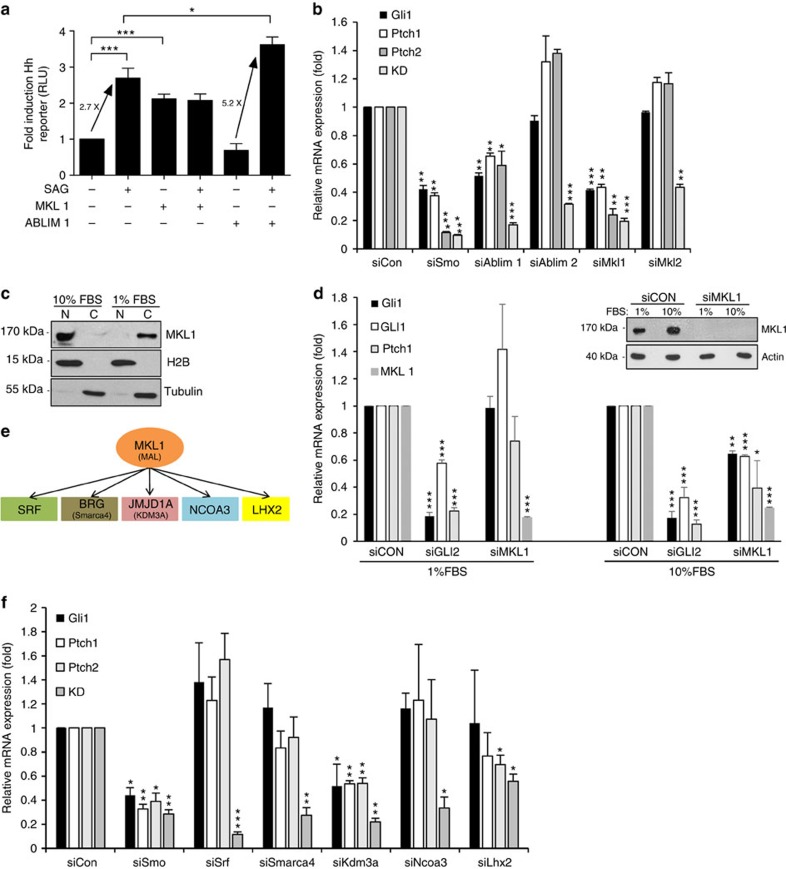
ABLIM and MKL1 are positive regulators of endogenous Hh signalling. (**a**) Endogenous Hh signalling in NIH3T3 cells (1% FBS) measured by means of a luciferase reporter. Cells were transiently transfected with the constructs shown and were subsequently left untreated or were treated with the SMO agonist SAG (mean of *n*=3±s.d.). (**b**) Hh target gene expression in MEF^[SHH]^ cells transfected with the siRNA constructs indicated. RNAi against *Smo* was used as a positive control. KD, knockdown efficiency of the respective gene compared with control siRNA (mean of *n*=3±s.d.). (**c**) Immunoblot of A549 cytoplasmic (c) and nuclear (n) fractions showing shuttling of MKL1 in response to serum concentrations. H2B, histone H2B (nuclear marker protein); tubulin (cytoplasmic marker protein). (**d**) Hh target gene expression in human A549 cells transfected with the siRNA constructs indicated. RNAi against *GLI2* was used as a positive control. KD, knockdown efficiency of the respective gene compared with control siRNA. The inset depicts the loss of MKL1 protein after RNAi-mediated knockdown (mean of *n*=3±s.d.). (**e**) Schematic diagram showing known nuclear MKL1 interactors. If different from the protein name, gene names are given in brackets. (**f**) Hh target gene expression in MEF^[SHH]^ cells transfected with the siRNA constructs indicated. RNAi against *Smo* was used as a positive control. KD, knockdown efficiency of the respective gene compared with control siRNA (mean of *n*=3±s.d.). *P<0.05; **P<0.005; ***P<0.0005 (Student’s *t*-test).

To further investigate the impact of ABLIM1 as well as MKL1 on the regulation of Hh signalling, we performed reporter experiments. As can be seen in Fig. 4a; Supplementary Fig. 4a, the SAG-induced stimulation of endogenous Hh signalling in NIH3T3 cells could be significantly increased by co-expression of ABLIM proteins. In addition, transfection of MKL1 was sufficient to trigger Hh signalling even in the absence of SAG (Fig. 4a). Surprisingly, overexpression of ABLIM1 did not show this effect. The Hh stimulatory role of ABLIM and MKL1 was further verified in knockdown experiments using MEF cells stably expressing the SHH ligand (MEF^[SHH]^ cells) (Fig. 4b). Both the knockdown of endogenous *Ablim1* as well as *Mkl1* reduced the expression of the endogenous Hh target genes *Gli1*, *Ptch1* and *Ptch2*, indicative of Hh pathway inhibition. The knockdown of *Ablim2* or *Mkl2* had no effect, presumably due to their low expression levels in MEF cells.

Taken together, our data postulate a model in which actin regulators (DYRK1A and ABLIM) modulate MKL1 shuttling through their effects on the F-actin cytoskeleton. A serum-dependent modulation of Hh signalling by MKL1 could also be shown in human A549 cells (Fig. 4c,d), where MKL1-directed siRNA is only effective under situations of nuclear MKL1 presence (that is, high serum concentration). In contrast, siRNA targeting *GLI2* inhibited Hh target gene expression irrespective of the serum concentration (Fig. 4d).

MKL1 itself is a transcriptional co-activator binding to several nuclear proteins, but not to GLI1 in our hands (Supplementary Fig. 4b). Therefore, we queried the role of the well characterized interaction partner SRF (Fig. 4e). Intriguingly, knocking down *Srf* mRNA in MEF cells had no impact on Hh signalling as measured by target gene expression (Fig. 4f), despite the fact that the expression of the well-established Srf target *Acta2* was almost completely abolished (Supplementary Fig. 4c). In light of the fact that an additional MKL1 interactor (BRG)^39^ had been reported as a Hh modulator^40^, we knocked down *Brg* (encoded by the *Smarca4* gene) expression but found no effect on endogenous Hh signalling either (Fig. 4f). Testing the importance of other reported MKL1 partners (JMJD1A, NCOA3 and LHX2)^41^,^42^ revealed that the Jumonji domain-containing histone demethylase 1A (JMJD1A, encoded by the *Kdm3a* gene) displayed a significant influence on Hh pathway activity (Fig. 4f). Taken together, we hypothesized that nuclear MKL1 exerts its positive role on Hh signalling through interaction with JMJD1A. In support of this assumption, the double knockdown of *Mkl1/Kdm3a* was similar to the single-gene knockdown with respect to its effect on Hh signalling (Supplementary Fig. 4d).

### Identification of a novel small-molecule GLI antagonist

**Figure 5:**
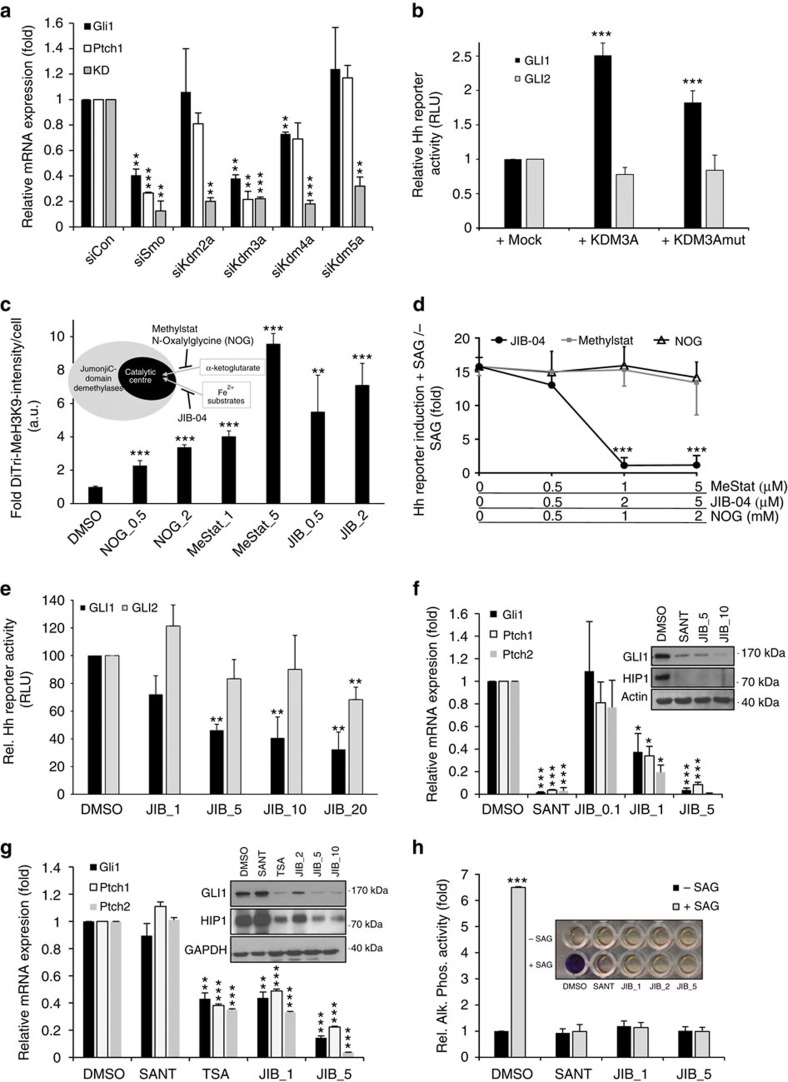
Identification of a small-molecule Jumonji inhibitor as a novel Hh antagonist. (**a**) Hh target gene expression in MEF^[SHH]^ cells transfected with the siRNA constructs indicated. RNAi against *Smo* was used as a positive control. KD, knockdown efficiency of the respective gene compared with control siRNA (mean of *n*=3±s.d.). (**b**) Luminometric Hh reporter assay in HEK293A cells transiently transfected with GLI1 or GLI2 (full length) and the indicated constructs. KDM3Amut: catalytically inactive mutant (KDM3A^H1122A^) (mean of *n*=4±s.d.). (**c**) Change in di/tri-methylated histone H3K9 in ShhL2 (5% FBS) cells after 48-h incubation with the indicated Jumonji inhibitors. MeStat, methylstat (1 and 5 μM); NOG, *N*-oxalyl-glycine (0.5 and 2 mM); JIB, JIB-04 (0.5 and 2 μM). Shown is the mean pixel intensity per cell of at least 200 cells (mean of *n*=3±s.d.). (**d**) Hh reporter assay in SAG-induced ShhL2 cells (5% FBS). The different concentrations of the compounds (48 h) are given on the *x* axis (mean of *n*=3±s.d.). (**e**) Luminometric Hh reporter assay in HEK293A cells transiently transfected with GLI1 or GLI2 (full length) and treated for 24 h with the indicated JIB concentrations, for example, JIB_10=JIB 10 μM. Mean of *n*=3±s.d. (**f**) Hh target gene expression (*Gli1, Ptch1* and *Ptch2*) in MEF^[SHH]^ cells treated with dimethylsulphoxide (DMSO), SANT (0.2 μM) or the indicated concentration of JIB-04 (for example, JIB_1, 1 μM) for 48 h. Inset: immunoblot of the same cells detecting GLI1 and HIP1 protein levels after compound exposure. Mean of *n*=3±s.d. (**g**) Hh target gene expression (*Gli1, Ptch1* and *Ptch2*) in *Sufu−/−* MEF cells treated with DMSO, SANT (0.2 μM), Trichostatin A (TSA) (0.5 μM) or the indicated concentration of JIB-04 (for example, JIB_1=1 μM) for 48 h. Inset: GLI1/HIP1 immunoblot of the same cells. Mean of *n*=3±s.d. TSA is an inhibitor of GLI transcription factors^62^ and was used as a positive control. (**h**) Osteogenic differentiation assay using C3H10T1/2 cells (5% FBS). AP, alkaline phosphatase. The inset shows a representative picture of the stained cells (AP activity in blue). Mean of *n*=3±s.d. *P<0.05; **P<0.005; ***P<0.0005 (Student’s *t*-test).

Next, we focused on the characterization of Hh pathway modulation by JMJD1A. First, we asked the question if other *Kdm* family members also regulate the Hh pathway or if the observed effect was specific to *Kdm3a*. To this end, we knocked down *Kdm2a*, *Kdm3a*, *Kdm4a* and *Kdm5a* mRNA expression in MEF^[SHH]^ cells and measured Hh target gene expression (*Gli1* and *Ptch1*). Interestingly, knockdown of *Kdm2a* or *Kdm5a* had no effect and only the knockdown of *Kdm3a* suppressed the expression of both Hh target genes, indicating that, at least in the cell type studied, a certain degree of *Kdm* preference exists (Fig. 5a). To elucidate whether the demethylase activity of JMJD1A is required for the regulation of Hh signalling, we performed Hh reporter assays using GLI1 and co-transfected JMJD1A (*KDM3A*). In agreement with a positive function in the Hh pathway, expression of *KDM3A* stimulated the transcriptional activity of GLI1 (Fig. 5b). Intriguingly, *KDM3A* expression had no significant effect on GLI2 function, suggesting a GLI1-selective mechanism. Moreover, transfection of an enzymatically inactive mutant (KDM3A^mut^; KDM3A^H1122A^ (ref. 43)) was also capable of potentiating GLI1 function, arguing that the observed impact on Hh signalling was not mediated through demethylation of histones (Fig. 5b) and that JMJD1A likely fulfills a scaffolding role in the Hh pathway.

Therefore, we hypothesized that small-molecule antagonists directly binding to Jumonji domain proteins might impose structural changes interfering with such a scaffolding function. Hence, we tested three structurally different Jumonji inhibitors, which either compete for binding with the co-factor α-ketoglutarate (methylstat and *N*-oxalyl-glycine) or bind to the Fe^2+^/catalytic center (JIB-04). Notably, all three inhibitors effectively increased the level of Di- and Tri-methylated histone H3 (lysine 9; Fig. 5c; Supplementary Fig. 5b), demonstrating inhibition of Jumonji-mediated histone demethylation. Strikingly, when these inhibitors were tested for their effect on Hh signalling, only one compound (JIB-04 (ref. 44)) was able to block Hh activity (Fig. 5d), indicating that structural effects on Jumonji proteins and not changes in histone methylation were most likely responsible for this outcome. Moreover, JIB-04 potently inhibited transfected GLI1 (with effects on GLI2 only at higher concentrations; Fig. 5e) and interfered with endogenous Hh target gene expression in MEF^[SHH]^ cells (Fig. 5f). We used these cells also for an additional investigation of a potential histone/chromatin/transcription-independent mechanism of JIB-04. Specifically, the kinetics of Hh target gene (*Gli1*) suppression induced by this compound were compared with other chromatin/transcription-based drugs, such as actinomycin D (blocking general transcription) or JQ1 (a BRD4-inhibitor involved in GLI-mediated transcription; ref. 45). Both the control inhibitors (actinomycin D and JQ1) reduced the level of *Gli1* mRNA with a half-time (*t*_1/2_) of 2–3 h (Supplementary Fig. 5c). In contrast, JIB-04 suppressed *Gli1* mRNA levels with a *t*_1/2_ of 5–6 h (Supplementary Fig. 5c), suggesting a different and potentially transcription-independent mode of Hh pathway inhibition by this compound.

In agreement with JMJD1A acting at the level of the GLI transcription factors, JIB-04 could also block signalling in *Sufu−/−* MEF cells, which harbour an activation of the Hh pathway downstream of SMO^24^ (Fig. 5g). Finally, to also investigate a Hh-driven physiological process, we turned to C3H10T1/2 mesenchymal progenitor cells, which undergo osteogenic differentiation on Hh pathway activation. As can be seen in Fig. 5h, SAG exposure induces the differentiation process as measured by the expression of alkaline phosphatase, a marker protein in bone differentiation. Exposure of the cells to JIB-04, however, completely blocked the SAG-induced alkaline phosphatase upregulation (Fig. 5h).

In summary, we could identify JMJD1A as a novel factor potentiating GLI1 activity, with little effect on GLI2. This stimulation is at least partly independent of JMJD1A’s histone demethylation ability. A small-molecule inhibitor of Jumonji enzymes functions as a novel antagonist of Hh signalling at the level of GLI1.

### JMJD1A (KDM3A) stabilizes GLI1 protein

**Figure 6:**
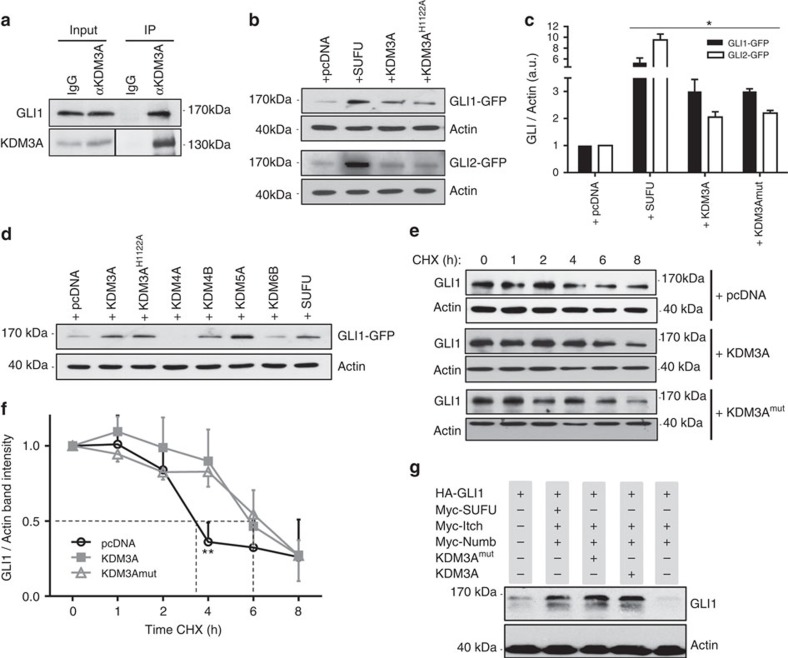
JMJD1A (KDM3A) stabilizes the GLI1 protein. (**a**) Co-immunoprecipitation experiment in Hek293T cells detecting an interaction between endogenous KDM3A and endogenous GLI1. (**b**) Co-transfection of GFP-tagged GLI1 or GLI2 (full length) and the indicated constructs in Hek293A cells. SUFU was included as positive control for GLI stabilization. Shown are representative results of three independent experiments. (**c**) Quantification of the results depicted in **b**. Shown is the mean GLI band intensity normalized to actin of *n*=3 experiments (±s.d.). (**d**) Western blot of Hek293A cell lysates after transfection of GLI1-GFP and the indicated constructs. Shown are representative results of three independent experiments. (**e**) Determination of the half-life of endogenous GLI1 protein in Hek293T cells transfected with empty vector control (pcDNA), KDM3A or its catalytically inactive mutant (KDM^mut^). CHX, cycloheximide (100 μg ml^−1^) for the indicated time periods. Shown are representative results of three independent experiments. (**f**) Quantification of the results depicted in **e**. Shown is the mean GLI band intensity normalized to actin of *n*=3 experiments (±s.d.). (**g**) Western blot result of co-transfection experiments in Hek293A cells. Shown is a representative result of three independent experiments. *P<0.05; **P<0.005; ***P<0.0005 (Student’s *t*-test).

In light of our finding that KDM3A expression enhances GLI1 activity, we were interested in the mechanism of this regulation. Interestingly, in co-immunoprecipitation experiments, we could detect an interaction between GLI1 and JMJD1A at endogenous expression levels (Fig. 6a). Moreover, co-transfection of *KDM3A* plasmid or its inactive mutant increased the protein amount of GLI1-GFP with less effect on GFP-tagged GLI2 (Fig. 6b,c). This protein stabilization effect could also be observed by co-expression of certain other KDM family members (*KDM4B* and *KDM5A*) but not of others (for example, *KDM4A* and *KDM6B*; Fig. 6d). Time-course analyses using the translational inhibitor cycloheximide revealed that expression of KDM3A (or its demethylase mutant) extended the half-life of endogenous GLI1 protein by ∼2.5 h, demonstrating a stabilizing effect (Fig. 6e,f).

Since GLI1 has been shown to be ubiquitinated and subsequently proteasomally degraded through the action of Itch and Numb^46^, we wondered whether KDM proteins protect GLI1 from Itch/Numb-mediated degradation. In fact, KDM3A (and its inactive mutant) proved very efficient in rescuing GLI1 from Itch/Numb-mediated degradation (Fig. 6g). In summary, we could demonstrate that JMJD1A (KDM3A) directly associates with GLI1, protecting it from proteasomal degradation, leading to a stabilized protein with a longer half-life.

### JIB-04 blocks GLI1 activation in human cancer cells

Hh/GLI signalling has been implicated in the aetiology of several cancers, including pancreatic and lung cancer, rhabdomyosarcoma and medulloblastoma^29^,^47^,^48^,^49^,^50^,^51^.

**Figure 7:**
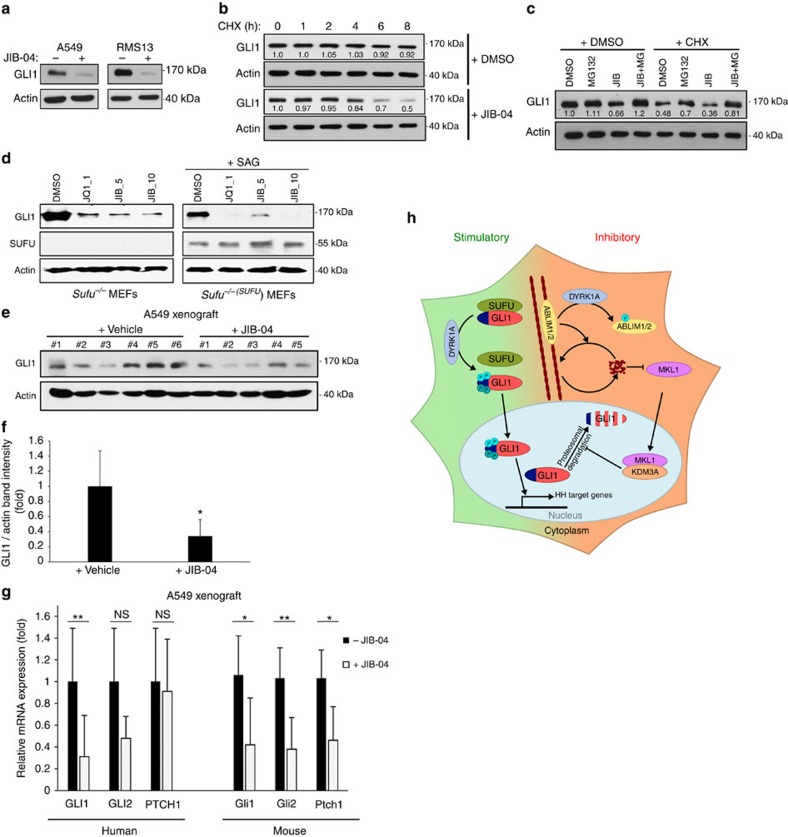
JIB-04 promotes GLI1 decay in human cancer cells. (**a**) Immunoblot detecting endogenous GLI1 protein in lysates of human lung carcinoma cells (A549) and human rhabdomyosarcoma cells (RMS13). Cells were treated with 10 μM JIB-04 for 24 h. Shown is a representative result of two independent experiments. (**b**) Endogenous GLI1 protein stability assay in RMS13 cells exposed to JIB-04 (10 μM) and cycloheximide (CHX, 100 μg ml^−1^) for the indicated time periods. Shown is a representative result of two independent experiments. Numbers indicate the GLI1 band intensity of the experiment shown normalized to actin. (**c**) Western blot detecting endogenous GLI1 protein in RMS13 cell lysates. Cells have been treated with the indicated compounds for 8 h. MG132: 10 μM; JIB-04: 10 μM. Shown is a representative result of three independent experiments. Numbers indicate the GLI1 band intensity of the experiment shown normalized to actin. (**d**) Western blot detecting endogenous GLI1 in *Sufu−/−* MEF cells and in *Sufu−/−* MEF cells stably expressing SUFU (*Sufu−/−*^[SUFU]^ cells). Cells were treated for 8 h with the indicated compounds. SAG: 100 nM; JIB-04: 1, 5, 10 μM. The BRD4 and GLI inhibitor JQ1 (ref. 45) was used as a positive control (1 μM). Shown is a representative result of three independent experiments. (**e**) GLI1 immunoblot of A549 xenograft samples receiving either solvent or JIB-04 (55 mg kg^−1^ by oral gavage two to three times weekly for 1 month). (**f**) Quantification of GLI1 western blot bands (normalized to actin) shown in **e**. (**g**) Murine and human Hh pathway gene expression in A549 xenograft samples as measured by species-specific qPCR (mean of *n*=7±s.d., significance calculated using two-way analysis of variance). (**h**) Scheme depicting the major findings of this manuscript. DYRK1A can stimulate GLI1 activity by direct phosphorylation (left side). In contrast, DYRK1A is able to repress GLI1 activity through an indirect mechanism involving the actin cytoskeleton and its regulators. *P<0.05; **P<0.005; ***P<0.0005 (Student’s *t*-test).

In line with our previous data of KDM3A as a GLI-stimulating factor, we found *Kdm3a* mRNA expression to be increased in two independent mouse models of Hh-driven medulloblastoma (Supplementary Fig. 6a,b). In addition, JIB-04 decreased cell growth and Hh target gene expression in pancreatic cancer cells (Supplementary Fig. 7a,b,c) and GLI1 protein levels in lung carcinoma as well as in rhabdomyosarcoma cells (Fig. 7a). JIB-04 treatment clearly decreased the protein stability of endogenous GLI1 (Fig. 7b) as well as exogenous GLI1 (Supplementary Fig. 7d). As such, JIB-04-induced GLI1 degradation could be rescued by co-application of the proteasome inhibitor MG132 (Fig. 7c). Moreover, JIB-04 reduced GLI1 protein levels independently of SUFU, another direct GLI-binding protein capable of GLI stabilization^52^,^53^,^54^ (Fig. 7d).

To investigate whether JIB-04 affects GLI1 levels also under *in vivo* conditions, we analysed samples derived from a previously reported lung cancer cell (A549) xenograft experiment in which JIB-04 led to significant growth inhibition^44^. As can be seen in Fig. 7e,f, JIB-04-exposed xenograft samples showed strongly reduced GLI1 protein levels. Furthermore, using species-specific primers, we could show that JIB-04 decreased Hh target gene expression in the human lung cancer cells as well as in the murine stroma (Fig. 7g). Taken together, JIB-04 promotes the degradation of GLI1 in several human cancer cell lines and inhibits Hh signalling *in vitro* and *in vivo*.

## Discussion

This work describes two DYRK1A-modulated regulatory arms within the mammalian Hh signalling cascade (Fig. 7g). On the basis of our data, we assume that the GLI1-stimulating mechanisms of DYRK1A might potentially be actin independent, whereas the GLI1-suppressive mechanism involves the actin cytoskeleton. The final decision about which signalling arm is executed might be cell-type specific or be dependent on the expression levels of DYRK1A and GLI1. An alternative and currently unproven hypothesis would entail GLI2 being involved in the cellular stimulation of the Hh pathway by DYRK1A whereas the reduction in GLI1 levels would be involved in the negative pathway regulation. However, DYRK1A-mediated stimulation of GLI1 was mostly observed under conditions of very high DYRK1A expression levels and in many cases in combination with transiently transfected GLI1 (which typically yields very high expression levels as well). Here DYRK1A phosphorylates two nuclear import clusters located within the N-terminal domain of GLI1, leading to enhanced nuclear import. Although the conditions of these experiments would suggest that DYRK1A-induced GLI1 stimulation would primarily occur in artificial transfection situations, we cannot rule out that endogenous situations exist in which DYRK1A would exert a positive role on Hh signalling (as, for example, in Supplementary Fig. 1c). However, in the vast majority of our experiments using endogenous Hh pathway situations *in vitro* and *in vivo*, we could observe a negative impact of DYRK1A on Hh signalling with ABLIM1/2, MKL1 and KDM proteins functioning as positive modulators. We believe that the observed negative role of DYRK1A on endogenous Hh signalling can very well explain the reduced Hh sensitivity observed in Down syndrome patients^10^,^11^.

Mechanistically, ABLIM proteins directly bind to DYRK1A and are phosphorylation substrates of the latter (Fig. 3a,b). Although further work is required, we conclude that DYRK1A-mediated phosphorylation functionally inactivates F-actin formation induced by ABLIM proteins. The regulation of the actin cytoskeleton subsequently impacts on the nucleo-cytoplasmic distribution of the transcriptional co-activator MKL1. Nuclear MKL1 stimulates Hh signalling in an SRF-independent manner, most likely through the presence of one of its direct interactors, the histone demethylase JMJD1A (ref. 41). Alternatively, MKL1 might act to recruit additional transcriptional co-activators such as histone acetyltransferases to the GLI complex.

With respect to the final effector of the DYRK1A/ABLIM/actin/MKL1 regulatory axis, JMJD1A, our data suggest a Hh pathway regulation that is independent of its histone demethylase activity. However, we should state that at this point we cannot fully rule out chromatin-related effects of JMJD1A or its inhibitor JIB-04, which could occur at later time points or through indirect mechanisms. Histone methylation-dependent effects can also not be fully excluded in long-term experiments such as cell growth assays. Nevertheless, our observations still suggest that JMJD1A fulfills a scaffolding role in the GLI transcriptional complex, a hypothesis underscored by our finding of a direct GLI1–JMJD1A interaction. JMJD1A protects GLI1 from Itch/Numb-mediated proteasomal decay, a phenomenon more pronounced in the case of GLI1 in comparison with GLI2. The mechanistic explanation for this selectivity needs further experimentation, but might involve the differential routes of degradation (Itch/Numb for GLI1 and Spop for GLI2 (ref. 52)). Alternatively, differential methylation of GLI proteins followed by recognition through Jumonji enzymes might account for differences in GLI1 versus GLI2 stabilization efficiencies. However, the surprising preference for GLI1 could explain the lack of a lethal Hh phenotype in *Mkl1* or *Kdm3a* knockout animals^55^,^56^ as *Gli1* null animals show no apparent developmental phenotype, a finding which is in contrast to the embryonically lethal knockout of the *Gli2* gene^57^. *Since Gli1* is dispensable for normal embryonic development^57^, but indispensable for tumour growth^58^, it represents a formidable drug target. In line with this concept, small-molecule Jumonji inhibition exerted strong anti-neoplastic effects *in vitro* and *in vivo* (ref. 44 and this manuscript). Although further work on this matter is required, we hypothesize that JIB-04 alters the protein conformation and thereby interferes with JMJD1A’s scaffolding role. In support of this hypothesis, we found that unrelated Jumonji domain antagonists were able to block histone demethylation, but did not impinge on Hh signalling.

In a wider perspective, our data suggest a regulatory role of the actin network on the Hh pathway. Given that the actin cytoskeleton is responsible for the mechanosensitivity of cells^59^, it will be interesting to learn if the extracellular environment can modulate Hh signalling. For instance, several pathological processes, such as fibrosis or carcinogenesis, are associated with increased tissue stiffness and with active Hh signalling^60^, suggesting a possible mutual crosstalk. However, in light of the fact that the actin cytoskeleton also regulates other signalling pathways, such as Hippo/YAP and that these pathways can also crosstalk to the Hh pathway^61^, more research is needed on this matter.

In conclusion, the data presented provide a mechanistic framework on the potential molecular reasons behind the reduced Hh sensitivity in Down patients^10^,^12^ and why some of the Down-related clinical syndromes could be ameliorated by addition of the SMO agonist SAG^11^. Furthermore, in light of our data showing that DYRK1A negatively regulates the oncogenic Hh pathway, it is well conceivable why this gene has been described as a tumour suppressor in, for example, medulloblastoma^20^. Most importantly, our results identify a novel and GLI1-selective Hh pathway antagonist that can serve to develop new targeted anti-Hh therapies.

## Methods

### Protein microarray

For the identification of DYRK1A protein substrates, we purchased the ProtoArray Human Protein Microarray v5.0 kinase substrate identification (KSI) kit from Invitrogen (#PAH0525065, containing ∼9,480 recombinant GST-tagged and baculovirus-expressed proteins in duplicate spots). The arrays were incubated with enzymatically active recombinant DYRK1A (Invitrogen, PV3785) and γ^33^P-ATP. The control slide received only radioactive ATP (no kinase). All technical procedures as well as the data analysis were according to guidelines given in the manual.

### Luciferase reporter assays

ShhL2 cells were plated in triplicate and were grown to full confluency in solid white 96-well plates with clear bottom. Subsequently, cells were treated in full growth medium with 100 nM SAG plus the indicated compounds for 48 h. Cells were lysed in Passive Lysis Buffer (Promega) and Firefly and Renilla luciferase activity were measured by an Orion L microplate luminometer (Berthold Detection Systems) using Beetle- and Renilla-Juice reagents (both PJK).

### Osteogenic differentiation assay

The mesenchymal progenitor cell line C3H10T1/2 was plated in triplicates and grown to full confluency in 96-well plates. Subsequently, cells were exposed to experimental compounds in full growth medium for 4 days. Afterwards, cells were lysed in Passive Lysis Buffer (Promega). A part of the lysate was used for protein quantification (Bio-Rad Protein Assay, Bio-Rad), while the remaining lysate was used for measuring alkaline phosphatase activity (Alkaline Phosphatase Blue Microwell Substrate, Sigma).

### Phalloidin staining and microscopy

Cells were transfected in culture with siRNA (35 nM, Dharmacon) or plasmid using RNAiMax (Life Technologies) or TransIT (Mirus Bio), respectively. Subsequently, cells were seeded on glass cover slips and fixed with 4% formaldehyde/PBS for 10 min at room temperature. After washing with PBS, cells were permeabilized with 0.1% Triton-X100/PBS at room temperature for 5 min. Following another wash step, actin filaments were stained with California Red-conjugated phalloidin (Biomol) for 20 min at room temperature. After washing with PBS and rinsing with H_2_O, the cells were covered with mounting medium (Vectashield). Microscopy was performed on a Leica AF6000 3D Deconvolution microscope system.

### Immunoblotting

Separation of lysates by SDS–polyacrylamide gel electrophoresis was followed by subsequent western blot analysis. SDS–polyacrylamide gel electrophoresis gels were blotted on Immobilon-polyvinylidene difluoride membranes (Millipore) and incubated with the respective primary antibody, followed by an horseradish peroxidase-coupled secondary antibody. Detection of the horseradish peroxidase signal was performed using Pierce ECL Western Blotting Substrate (Thermo Scientific) according to the manufacturer’s protocol. Antibodies used are listed in Supplementary Data 1, and uncropped versions of most important western blots are in Supplementary Fig. 8.

### siRNA transfection, RNA preparation, cDNA synthesis and qPCR

In cases of siRNA transfection, cells were transfected with 35 nM siRNA (Dharmacon) using RNAiMax (Invitrogen). Total RNA was extracted using NucleoSpin RNA II kit (Macherey-Nagel) according to the manufacturer’s protocol. Complementary DNA (cDNA) synthesis of 1 μg total RNA was performed using iScript cDNA Synthesis Kit (Bio-Rad) following the manufacturer’s guidelines. Quantitative PCR (qPCR) reactions were performed using the Absolute QPCR SYBR Green Mix (ABGene). qPCR reactions were performed on 96-well qPCR plates (ABGene) using either the Mx3000P or Mx3005P qPCR systems (Agilent). Results were calculated as relative mRNA expression (2^ΔΔCt^). Data were obtained from at least three independent experiments and are shown as the mean±s.d. Primer sequences can be found in Supplementary Data 1.

### G-actin versus F-actin fractionation assay

The actin fractionation was performed according to the ‘G-actin/F-actin *in vivo* Assay Kit’ (Versions 1.1 and 3.5) from Cytoskeleton Inc. (Denver, USA).

### Xenograft assay

The A549 xenograft experiment has been described in ref. 44. Briefly, 4–6-week-old female nude mice were injected subcutaneously with five million A549 cells and tumours were allowed to grow for 2–3 weeks. When tumours reached ∼200 mm^3^, therapy was started in weight and tumour volume matched pairs (*n*=7 for each treatment group). Drug or vehicle was administered three times per week by oral gavage in 12.5% Cremophor EL, 12.5% dimethylsulphoxide as an aqueous suspension at 55 mg kg^−1^ for 5 weeks. At the end point, mice were euthanized by CO_2_ asphyxiation and cervical dislocation, and tumours were collected for further analyses.

### Statistical analysis

Unless otherwise stated, data are presented as the mean of three independent experiments±s.d. Statistical significance was calculated by applying a two-tailed Student’s *t*-test. *P<0.05; **P<0.005; ***P<0.0005 except for statistical calculation of the *in vivo* gene expression data in A549 xenograft samples where two-way analysis of variance plus Tukey’s correction for multiple testing was performed.

### Harmine treatment of animals

Twelve-week-old wild-type (wt; *n*=10) or LSL-Kras^G12D/+^, LSL-Trp53^R172H/+^, Pdx1-Cre mice (ref. 27; KPC; *n*=10) were injected once daily intraperitoneally; 5 days per week, for 8 weeks with 200 μl of a 30 mg kg^−1^ harmine (TCI) solution dissolved in 45% 2-hydroxypropyl-ß-cyclodextrin (Sigma) in PBS. At the end of the treatment period, mice were killed by cervical dislocation and pancreata were harvested for histology and protein isolation. Non-harmine-receiving age-matched animals (wt (*n*=5) and KPC (*n*=9)) were used for comparison. All animal experiments were approved by regional state authorities (Regierungspräsidium Giessen).

## Additional information

**How to cite this article:** Schneider, P. *et al.* Identification of a novel actin-dependent signal transducing module allows for the targeted degradation of GLI1. *Nat. Commun.* 6:8023 doi: 10.1038/ncomms9023 (2015).

## Supplementary Material

Supplementary Figures, Supplementary Table, Supplementary Methods and Supplementary References Supplementary Figures 1-8, Supplementary Table 1, Supplementary Methods and Supplementary References

Supplementary Data 1 Primary antibodies and siRNA sequences
