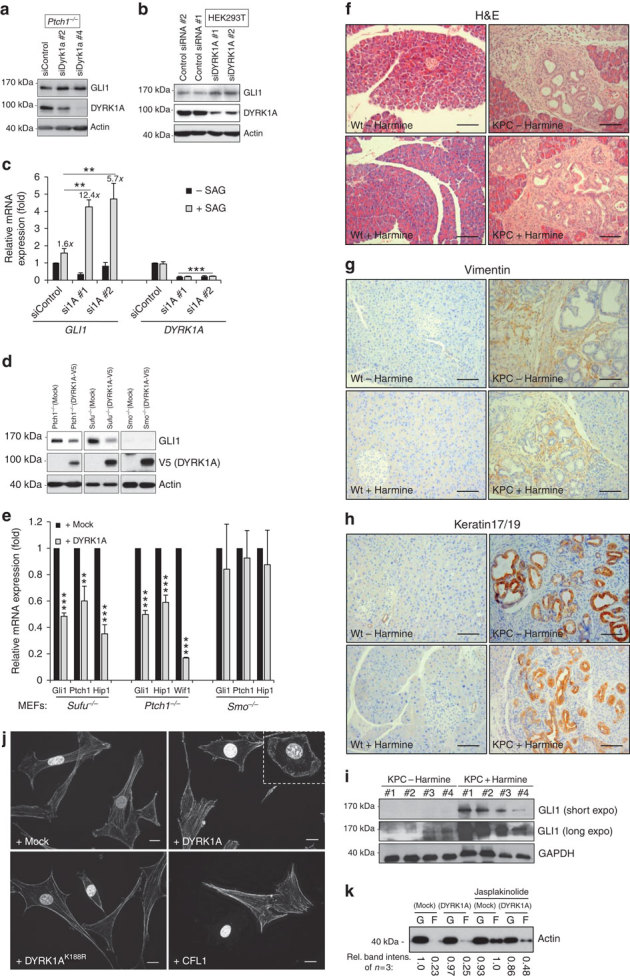# Correction: Corrigendum: Identification of a novel actin-dependent signal transducing module allows for the targeted degradation of GLI1

**DOI:** 10.1038/ncomms9741

**Published:** 2015-10-26

**Authors:** Philipp Schneider, Juan Miguel Bayo-Fina, Rajeev Singh, Pavan Kumar Dhanyamraju, Philipp Holz, Aninja Baier, Volker Fendrich, Annette Ramaswamy, Stefan Baumeister, Elisabeth D. Martinez, Matthias Lauth

10.1038/ncomms9023

In Fig. 2j of this Article, the image in the lower left panel was inadvertently duplicated from the upper left panel during the final stages of manuscript preparation. The correct version of the figure appears below.

**Figure 2:**