# Supplementary material for: Identification of a novel actin-dependent signal transducing module allows for the targeted degradation of GLI1
Source: Nat Commun. 2015 Aug 27;6:8023. doi: 10.1038/ncomms9023 (PMC4552080; doi:10.1038/ncomms9023)
Supplement: Supplementary Figures, Supplementary Table, Supplementary Methods and Supplementary References — Supplementary Figures 1-8, Supplementary Table 1, Supplementary Methods and Supplementary References [file ncomms9023-s1.pdf]

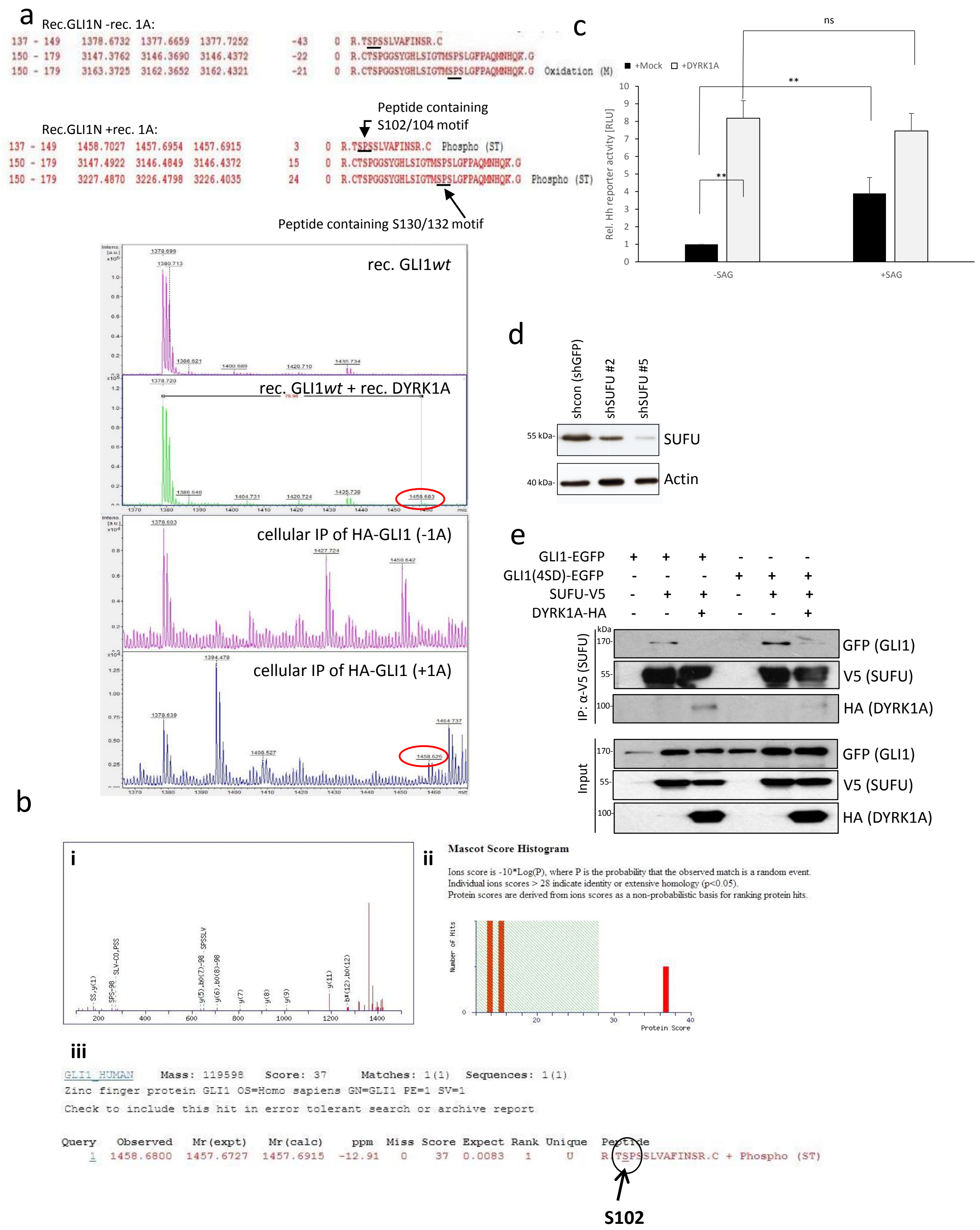

Supplementary Figure 1: DYRK1A phosphorylates the N-terminus of GLI1

(a) Upper panel: MALDI results from *in vitro* kinase reaction of GLI1 N-terminal domain with or without recombinant DYRK1A. Lower panel: Sections of the MALDI mass spectra of tryptic peptides of either recombinant GLI1 N-terminal domain (upper two panels) or cell-derived full length GLI1 (lower two panels). The peak at m/z 1378.7 (arrows) corresponds to the peptide aa137-149 of recombinant GLI1-Nterm *wt* (aa101-113 of the full length protein) with the sequence TSPSSLVAFINSR.

Phosphorylation of GLI1 with the kinase DYRK1A resulted in the appearance of an additional mass of 1458.8 (m/z) which corresponds to the peptide aa137-149 (aa103-113) phosphorylated at a single serine or the threonine residue, respectively. This phosphorylation-mediated shift in mass was not observed in a parallel experiment using recombinant GLI1-N-terminus in which S102/104 are mutated (not shown).

(b) The phosphorylation site of recombinant GLI1-Nterm was confirmed by MS/MS. (i) MS/MS spectrum of the fragmented peptide aa137-149 (1458.7 m/z) of the recombinant GLI1 (equaling aa 101-113 of full-length GLI1). (ii+iii) Bioinformatic analysis of the generated ion-series using Biotools™ and MASCOT-Server ™ software identified phosphorylated S138 which corresponds to S102 of the full length GLI1.

(c) Luminometric Hh reporter assay measuring endogenous pathway activity in NIH3T3 cells (mean of n=3 ±StDev). In addition to the Hh reporter plasmids, cell received empty vector (mock) or DYRK1A expression plasmid.

(d) Western blot of Hek293T cells transfected with control shRNA plasmid or shRNA constructs targeting SUFU as used in figure 1F.

(e) Co-immunoprecipitation experiment in Hek293T cells. Shown is a representative result of two independent experiments.

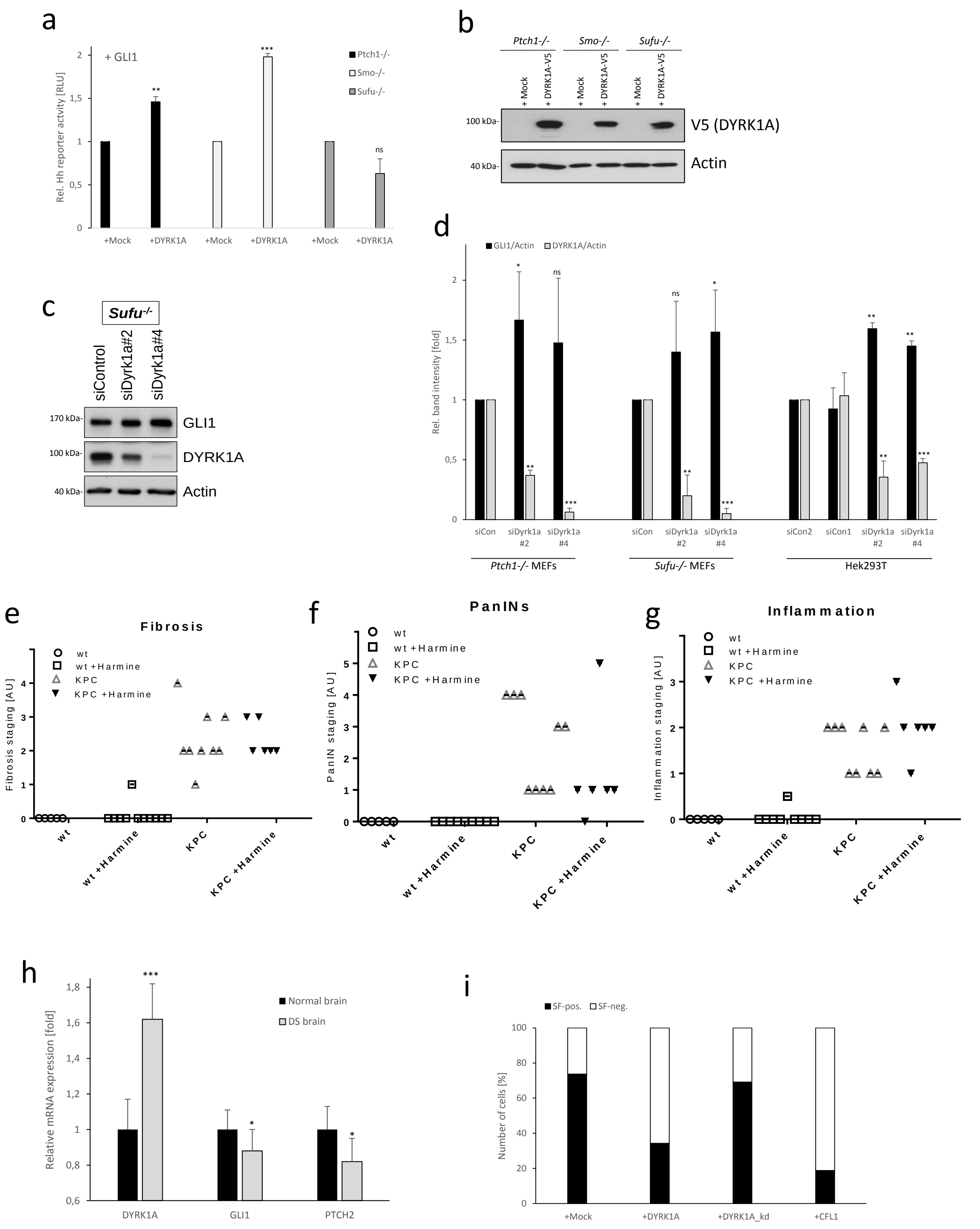

Supplementary Figure 2: DYRK1A reduces endogenous Hh signaling

(a) Luminometric Hh reporter assay measuring the activity of transfected GLI1 in different MEF cell lines (mean of n=3-4 ±StDev). Statistical significance calculated compared to the corresponding mock control.

(b) Western blot showing comparable expression of V5-tagged DYRK1A in transfected cells depicted in panel (A).

(c) Endogenous GLI1 and DYRK1A protein expression in siRNA-transfected *Sufu-/-* MEFs (1 % FBS).

(d) Quantification of n=3 (±StDev) actin-normalized DYRK1A and GLI1 Western blot band intensities. Representative examples are shown in figures 2A, 2B and S2C.

(e) Fibrosis scoring of control- versus Harmine-treated KPC pancreata on a scale of 0 (no fibrosis) to 4 (extensive fibrosis).

(f) PanIn staging of control- versus Harmine-treated KPC pancreata. Scale: 0 (no PanIn); 1 (PanIn1A); 2 (PanIn1B); 3 (PanIn2); 4 (PanIn3); 5 (PDAC).

(g) Inflammation scoring of control- versus Harmine-treated KPC pancreata on a scale of 0 (no inflammation) to 4 (extensive inflammation).

(h) Gene expression (as measured by microarray) of *DYRK1A* (probe ID: 209033_s_at), *GLI1* (206646_at) and *PTCH2* (221292_at) in normal (n=8) and in Down syndrome (DS, n=7) post-mortem brain tissue (dorsolateral prefrontal cortex).

(i) Quantification of the results shown in figure 2F. Scored was the number of stress-fiber (SF)-positive and –negative NIH3T3 cells after transient transfection of the indicated plasmids (identified by cotransfection of H2B-GFP). Shown is the mean of two independent experiments with at least 50 cells counted.

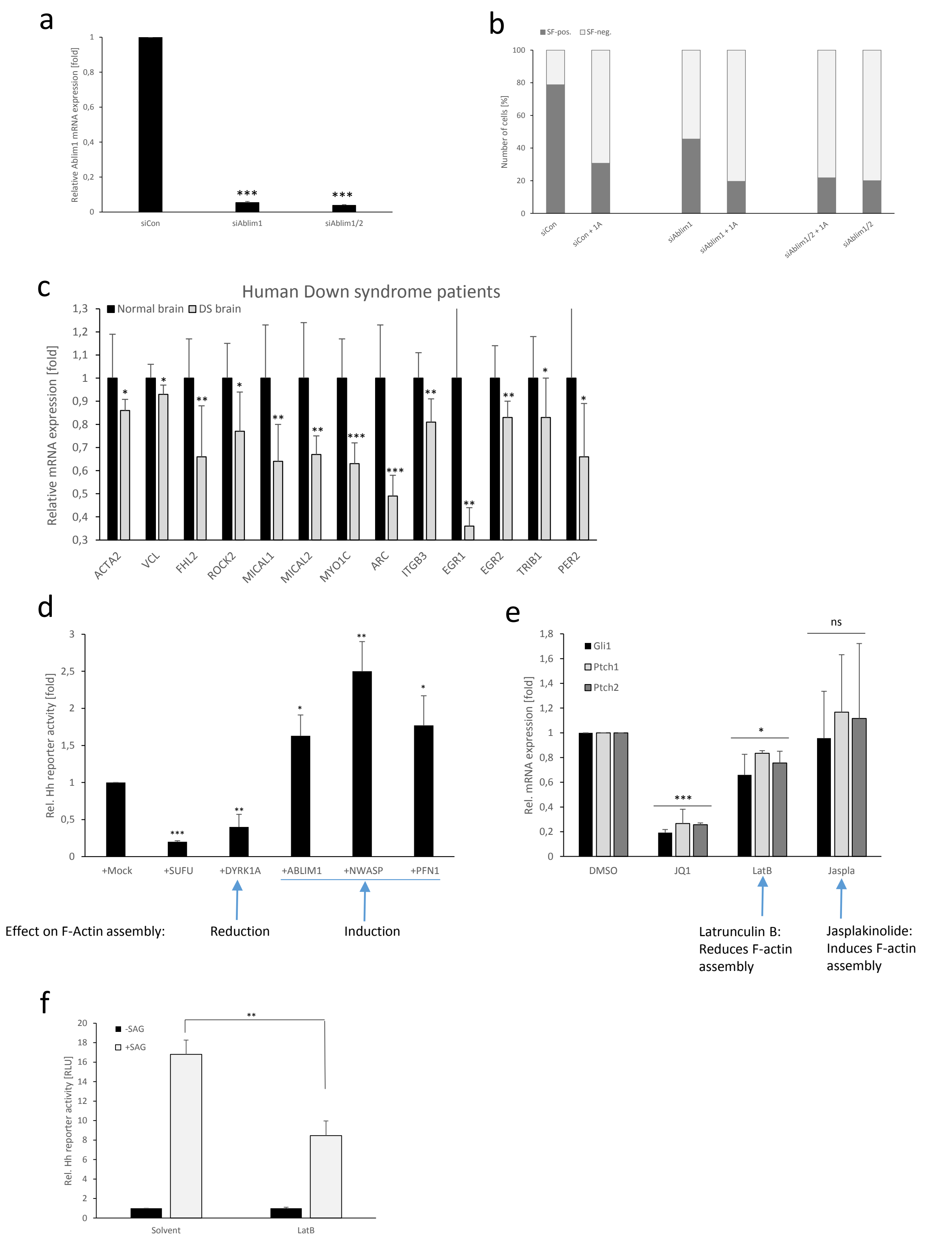

Supplementary Figure 3: ABLIM and Actin function in mammalian Hh signaling

(a) Verification of *Ablim1* knock-down in NIH3T3 cells used for microscopy in figure 3E. Endogenous *Ablim2* mRNA expression was not detectable.

(b) Quantification of the results shown in figures 3J and 3K. Scored was the number of stress-fiber (SF)-positive and –negative NIH3T3 cells after combined transient

transfection of siAblim1/siAblim1+2 alone or together with DYRK1A expressionplasmids (identified by cotransfection of H2B-GFP). Shown is the mean of two independent experiments with at least 50 cells counted.

(c) MAL/SRF target gene expression (as determined by microarray) in normal (n=8) and in Down syndrome (DS, n=7) brain tissue (dorsolateral prefrontal cortex). Probes: *ACTA2* (probe ID: 215787_at), *VCL* (200930_s_at), *FHL2* (202949_s_at), *ROCK2* (202762_at), *MICAL1* (218376_s_at), *MICAL2* (212472_at), *MYO1C* (214656_x_at), *ARC* (210090_at), *ITGB3* (204628_s_at), *EGR1* (201694_s_at), *EGR2* (205249_at), *TRIB1* (202241_at) and *PER2* (205251_at). Target genes were taken from [1, 2, 3, 4, 5].

(d) Luminometric Hh pathway reporter assay measuring endogenous signaling in *Sufu-/-* MEFs transfected with the indicated constructs (mean of n=3 ±StDev). The net effect of these actin regulators on F-actin assembly is given below.

(e) Hh target gene expression (*Gli1, Ptch1, Ptch2*) in *Sufu-/-* MEFs exposed to solvent (DMSO), JQ1 (1 µM, positive control), Latrunculin B (LatB, 1 µM) or Jasplakinolide (Jaspla, 1 µM) for 16 h.

(f) Luminometric Hh pathway reporter assay measuring endogenous signaling in ShhL2 cells (5 % FBS) treated with solvent or LatrunculinB (LatB, 1 µM) for 24 h (mean of n=3 ±StDev). For a better comparison, both '–SAG' samples were set to 1.

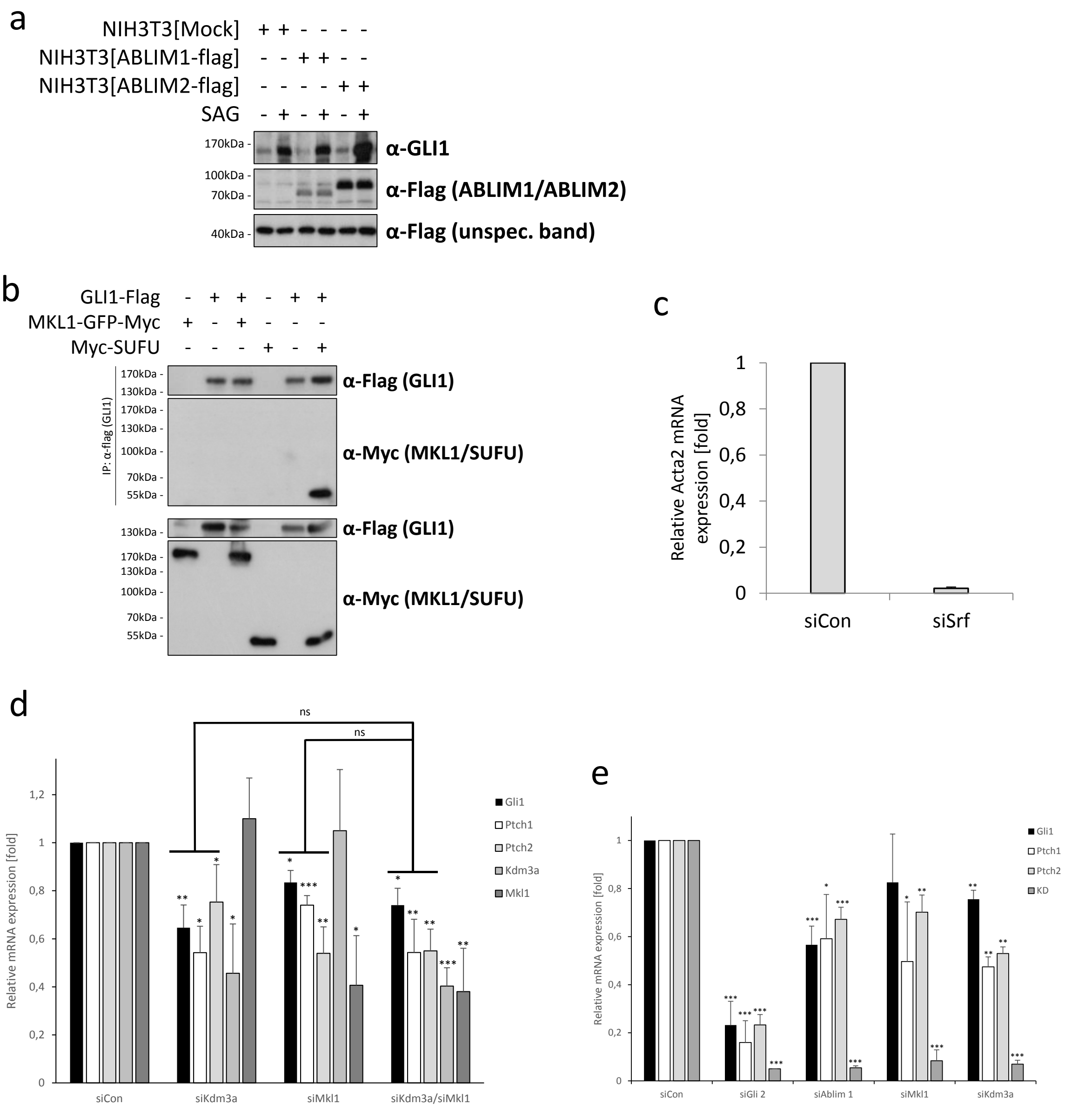

Supplementary Figure 4: Functional analysis of MKL1 and SRF

(a) Western blot analysis of NIH3T3 cells stably expressing ABLIM1 or ABLIM2. An unspecific band obtained with the Flag antibody was used as loading control.

(b) Lack of co-immunoprecipitation of MKL1 (MAL-GFP-Myc) and Flag-tagged GLI1 in Hek293T cells. As a positive control the SUFU-GLLI1 interaction was shown. A representative blot of two independent experiments is shown.

(c) Expression of the *Acta2* gene upon transfection of either control siRNA or *Srf*-specific RNAi into MEF$^{[SHH]}$ cells (mean of n=3 ±StDev).

(d) Hh pathway target gene expression (*Gli1, Ptch1, Ptch2*) in MEF$^{[SHH]}$ cells transfected with siRNA (final total concentration 70 nM; in single transfections, 35 nM target siRNA plus 35 nM control siRNA was used) (mean of n=3 ±StDev).

(e) Hh pathway target gene expression (*Gli1, Ptch1, Ptch2*) in *Sufu-/-* MEFs transfected with the indicated siRNAs. KD = Knock-down efficiency of the respective gene. Shown is the mean of n=3 ±StDev.

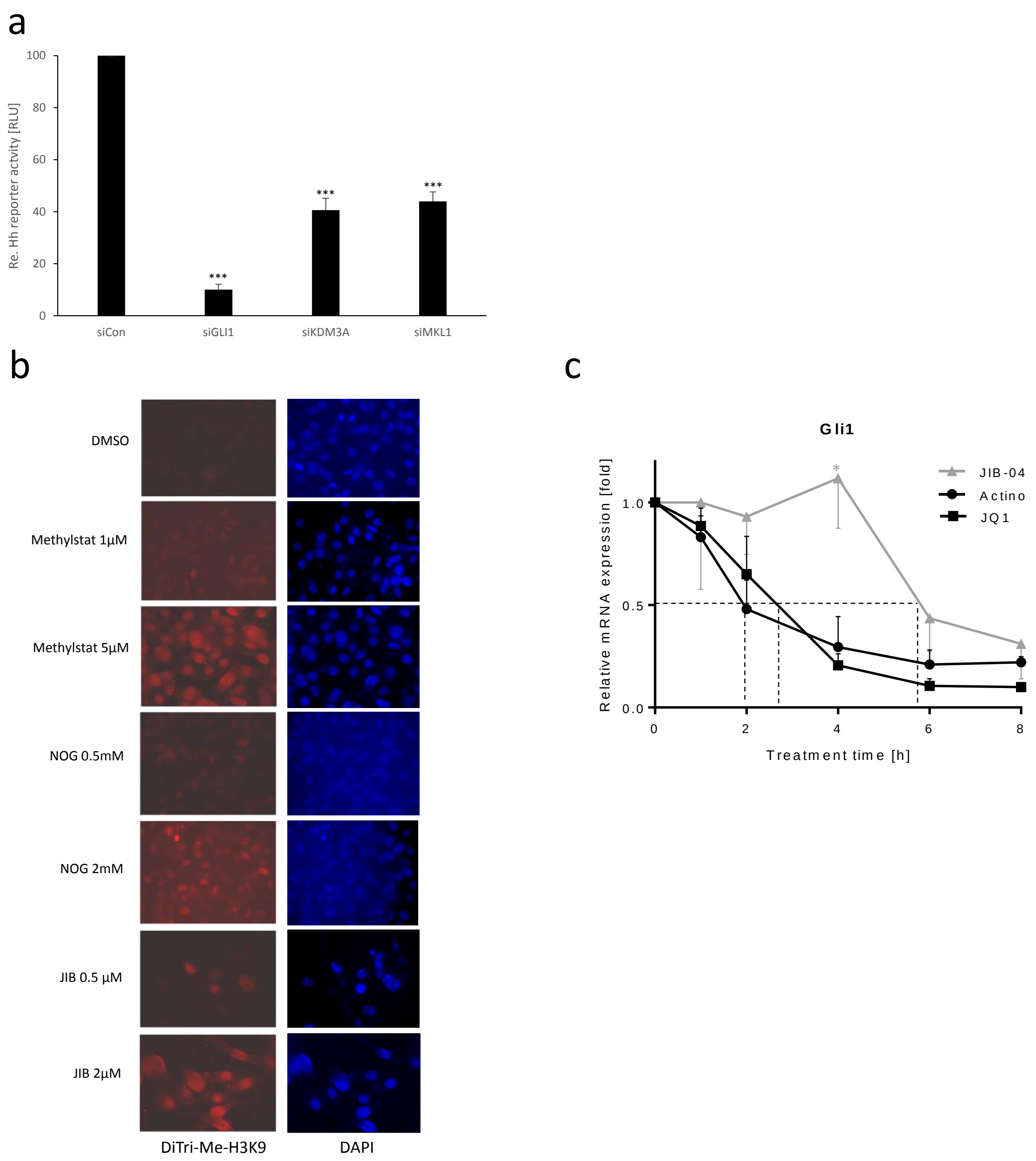

Supplementary Figure 5: Changes in histone methylation upon Jumonji inhibition

(a) Luminometric Hh reporter assay in GLI1-transfected Hek293A cells. In addition to the GLI1 expression plasmid, cells received the indicated siRNA sequences (mean of n=3 ±StDev). GLI1 siRNA was included as a positive control.

(b) ShhL2 cells were treated with Jumonji antagonists for 48 h and the levels of Di/Tri-methylation of H3K9 (red) were assessed by immunofluorescence. Blue staining indicates nuclei (DAPI). All pictures were taken using similar microscope settings.

(c) Time-course experiment in MEF[SHH] cells exposed to the indicated compounds (Actino=ActinomycinD (100 µg/ml); JQ1 (1 µM); JIB-04 (5 µM)). Shown is the mean expression of *Gli1* mRNA as measured by qPCR (n=3 ±StDev).

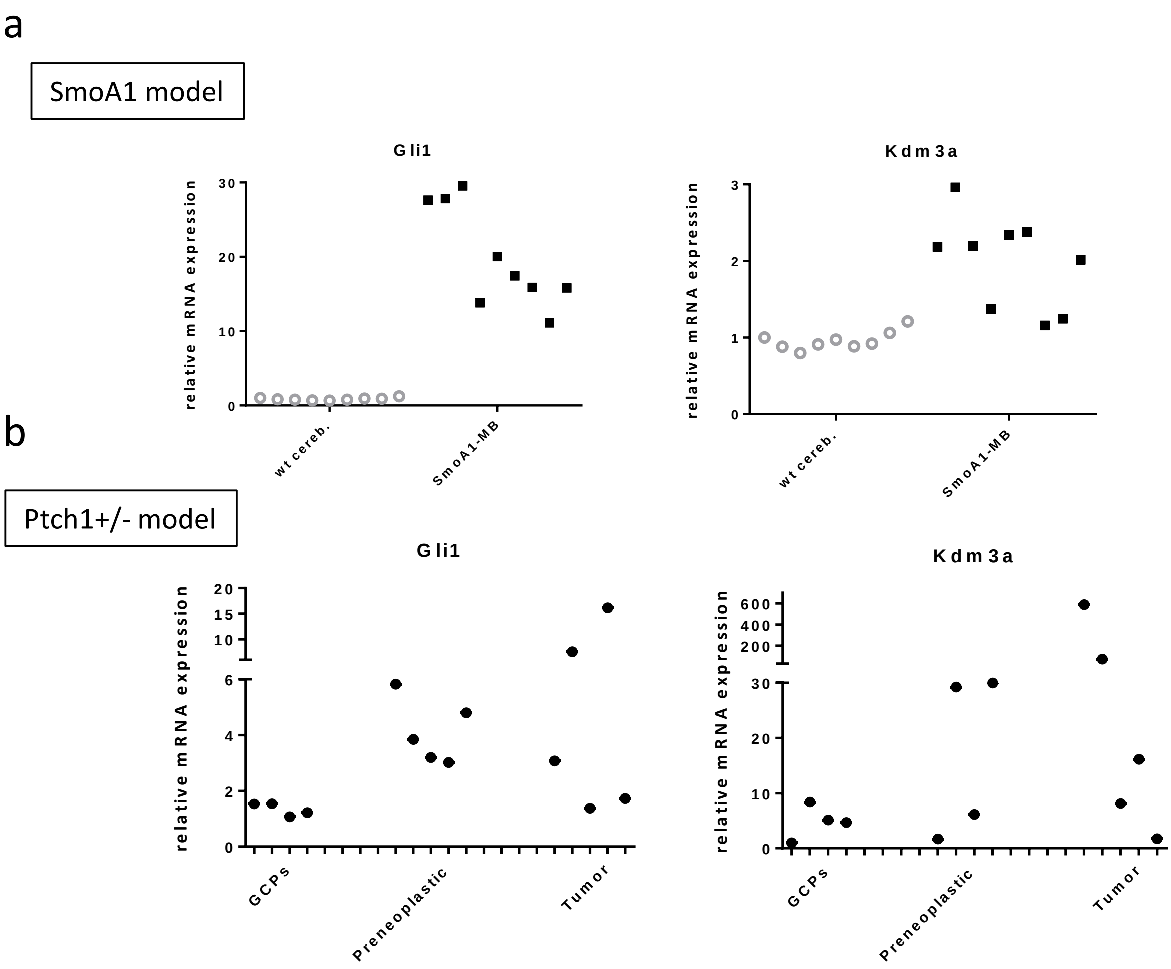

Supplementary Figure 6: *Kdm3a* expression in two different MB mouse models

(a) *Gli1* and *Kdm3a* mRNA expression as measured by qPCR in normal adult (wt, wildtype) cerebellum or in SmoA1-induced medulloblastoma (MB) (whole tissue).

(b) *Gli1* and *Kdm3a* mRNA expression as measured by microarray analysis of isolated primary cells. GCPs = Granule cell precursor cells harvested from P7 *Ptch1+/-* animals; preneoplastic = cerebellar cells isolated from 6-week old *Ptch1+/-* mice; Tumor = cells isolated from late stage MB tumors (10-25-week old *Ptch1+/-* animals with signs of MB). Reference: [6].

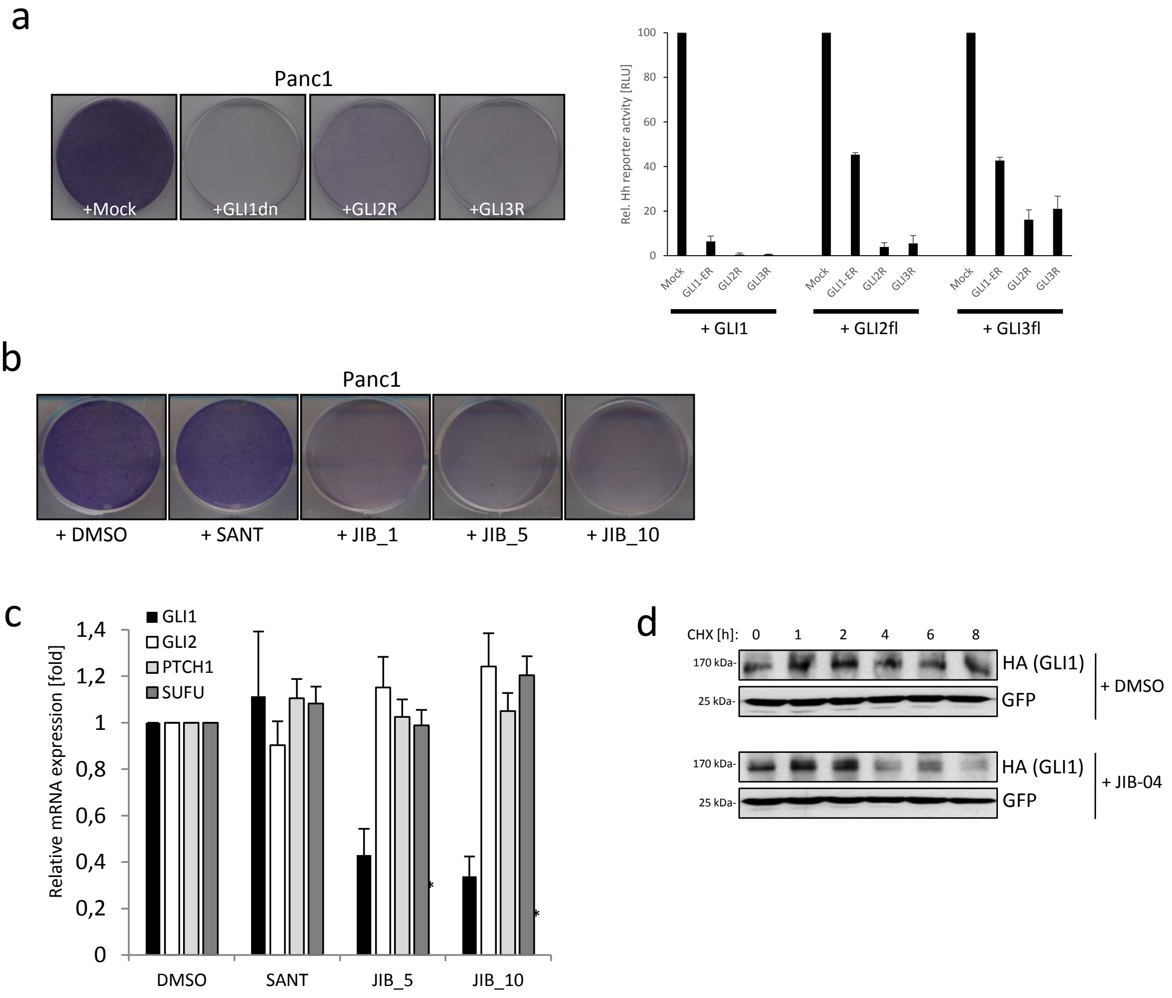

Supplementary Figure 7: JIB-04 represses GLI1 expression in pancreatic cancer cells

(a) Left: Interference with endogenous GLI function by transfection of dominant negative GLI constructs exerts strong inhibitory effects on cell proliferation. Shown are Panc1 cells transfected with the indicated constructs, followed by Blasticidin selection to enrich for transfected cells and stained after 1.5-2 weeks with Giemsa (blue). GLI1dn = dominant negative GLI1; GLI2R/GLI3R = Repressor variants of GLI2/GLI3.
Right: Verification of the dominant negative GLI constructs used in the left panel by means of a Hh reporter assay in Hek293T cells transfected with the indicated constructs. GLI1dn represents a fusion of full-length human GLI1 with the ligand-binding domain of the estrogen receptor (GLI1-ER), which strongly interferes with wild-type GLI1 function. GLI2R (aa1-676) and GLI3R (aa1-700) are truncated constructs representing the repressor versions of human GLI2 and GLI3, respectively. GLI2fl/GLI3fl=GLI2/3 full-length constructs.

(b) Treatment of Panc1 cells with JIB-04 phenocopies the effects of GLI inhibition as seen in (A). Note that the SMO antagonist SANT has no effect on these cells, arguing for downstream pathway activation. The concentration of JIB-04 is given in [µM] as e.g.: JIB_1 = JIB-04: 1 µM.

(c) Expression of Hh pathway components by qPCR of Panc1 cells treated with the indicated compounds for 48 h. Note that the expression of the non-Hh target gene *SUFU* is not affected by JIB-04, arguing against a general suppression of transcription.

(d) Immunoblots of lysates from Hek293A cells transfected with HA-GLI1 and EGFP expression plasmids. Both constructs are CMV-promoter-driven. Subsequently, cells were split and treated with Cycloheximide (100 µg/ml) for the indicated times plus either DMSO or JIB-04 (10 µM).

a DYRK1A overexpression dampens endogenous Hh signaling in Sufu-/- MEFs (Fig. 2d):

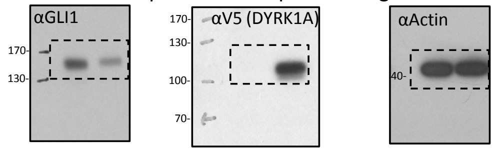

b Hh pathway inhibition by JIB-04 in Sufu-/- MEFs (Fig. 5g):

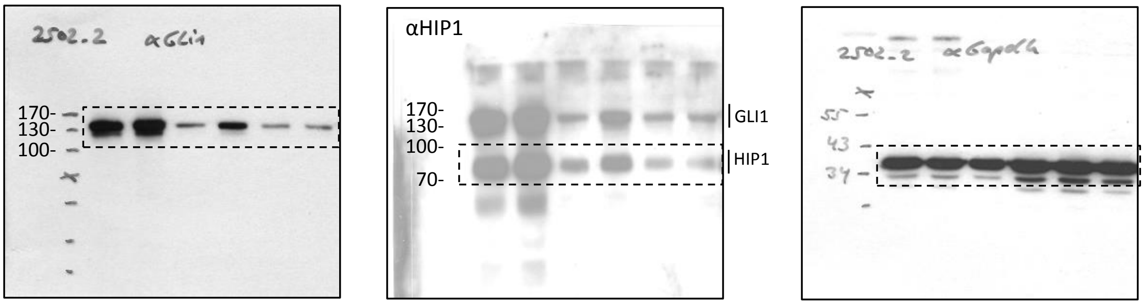

c KDM3a expression stabilizes GLI1-GFP with less effect on GLI2-GFP (Fig. 6b):

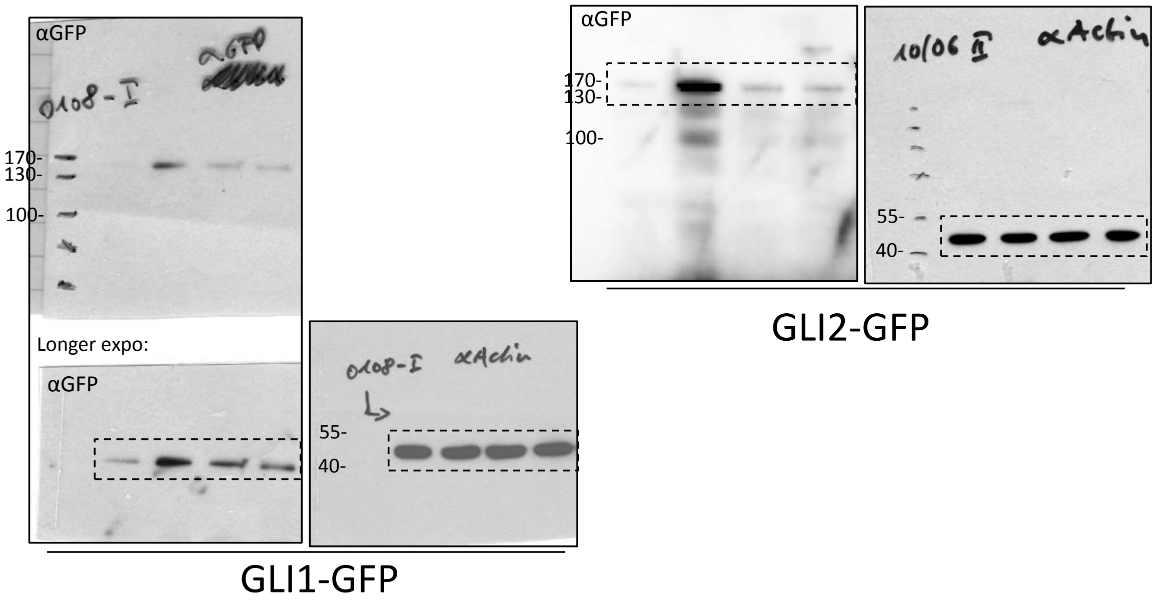

d JIB-04 destabilizes endogenous GLI1 protein in human cancer cells (Fig. 7b):

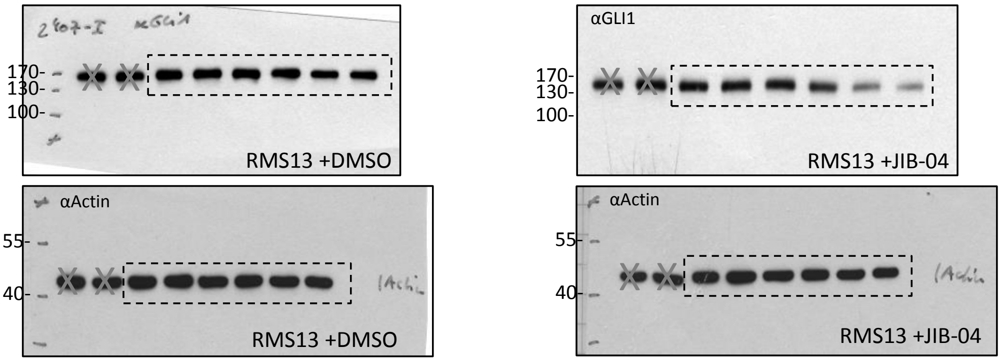

Supplementary figure 8: Original and uncropped scans of selected important Western blots

(a) Overexpression of DYRK1A dampens endogenous Hh signaling (Fig. 2d in the main manuscript). Sufu-/- MEFs were chosen as an example.
(b) Small molecule inhibition of Jumonji demethylases by JIB-04 blocks Hh target gene expression in Sufu-/- cells (Fig. 5g in the main manuscript).
(c) Expression of KDM3A stabilizes co-transfected GLI1-GFP (Fig. 6b) in the main manuscript).
(d) JIB-04 destabilizes endogenous GLI1 protein in human cancer cells (RMS13) (Fig. 7b in the main manuscript).

Supplementary Table 1

DYRK1A Substrates -Protein Microarray

Database ID	Block	Row	Column	Description	signal strength<br>1A
NM_020898.1	30	20	3	calcium binding and coiled-coil domain 1 (CALCOCO1)	XX
NM_004454.1	43	20	19	ets variant gene 5 (ets-related molecule) (ETV5)	XX
NM_005197.2	40	19	13	Forkhead box protein N3	XX
NM_004593.1	31	17	3	splicing factor, arginine/serine-rich 10 (transformer 2 homolog, Drosophila) (SFRS10)	XX
NM_144767.3	35	7	15	A kinase (PRKA) anchor protein 13 (AKAP13), transcript variant 3	X
BC002448.2	22	6	15	actin binding LIM protein 1 (ABLIM1)	X
NM_032204.3	23	4	15	activating signal cointegrator 1 complex subunit 2 (ASCC2)	X
NM_016638.1	18	15	3	ADP-ribosylation factor-like protein 6-interacting protein 4	X
BC015569.1	41	12	19	ADP-ribosylation factor-like protein 6-interacting protein 4	X
NM_153207.2	23	5	1	AE binding protein 2 (AEBP2)	X
NM_145865.1	19	7	5	ankyrin repeat and sterile alpha motif domain containing 4B (ANKS4B)	X
BC000238.1	22	13	13	ankyrin repeat and zinc finger domain containing 1 (ANKZF1)	X
BC043394.1	20	9	11	ankyrin repeat domain 17 (ANKRD17)	X
BC067773.1	1	6	13	arginine/serine-rich coiled-coil 2 (RSRC2)	X
BC010357.1	41	2	21	Arginine/serine-rich coiled-coil protein 1	X
XM_375456.2	18	8	3	Ataxin-7-like protein 3	X
BC044586.1	18	21	13	ATP-dependent RNA helicase DHX8	X
BC047327.1	26	21	11	ATP-dependent RNA helicase DHX8	X
NM_003600.1	44	9	17	aurora kinase A (AURKA), transcript variant 2	X
NM_022140.2	36	7	7	Band 4.1-like protein 4A	X
PV3371	17	15	19	BMX non-receptor tyrosine kinase (BMX), transcript variant 2	X
NM_020235.2	23	19	21	bobby sox homolog (Drosophila) (BBX)	X
NM_020836.2	24	9	1	brain-enriched guanylate kinase-associated homolog (rat) (BEGAIN)	X
NM_004656.2	34	5	17	BRCA1 associated protein-1 (ubiquitin carboxy-terminal hydrolase) (BAP1)	X
BC012576.1	31	7	21	bromodomain adjacent to zinc finger domain, 2B, mRNA (cDNA clone IMAGE:4290975), complete cds.	X
BC036335.1	19	20	21	BTB (POZ) domain containing 12 (BTBD12)	X
BC009961.1	31	15	5	BTB/POZ domain-containing protein KCTD17	X
PHC1475	28	16	5	C-C motif chemokine 21	X
NM_000723.3	16	4	21	calcium channel, voltage-dependent, beta 1 subunit (CACNB1), transcript variant 1	X
NM_024692.3	47	8	7	CAP-GLY domain containing linker protein family, member 4 (CLIP4)	X
BC032851.2	19	2	17	Cas-Br-M (murine) ecotropic retroviral transforming sequence b (CBLB)	X
BC008877.2	28	19	5	Caspase recruitment domain-containing protein 9	X
NM_006449.2	39	12	19	CDC42 effector protein (Rho GTPase binding) 3 (CDC42EP3)	X
BC011600.1	7	5	1	cDNA clone IMAGE:3050953, **** WARNING: chimeric clone ****	X
BC014452.1	39	16	13	cDNA clone IMAGE:4903661, complete cds	X
BC005383.1	19	4	9	centrin, EF-hand protein, 3 (CDC31 homolog, yeast) (CETN3)	X
NM_001819.1	42	13	19	chromogranin B (secretogranin 1) (CHGB)	X
BC022964.1	17	4	15	chromosome 1 open reading frame 107 (C1orf107)	X
NM_024319.1	37	13	19	chromosome 1 open reading frame 35 (C1orf35)	X
NM_014042.1	1	3	7	chromosome 11 open reading frame 51 (C11orf51)	X
BC051031.1	38	16	19	chromosome 11 open reading frame 74 (C11orf74)	X
NM_032848.1	30	15	11	chromosome 12 open reading frame 52 (C12orf52)	X
BC065370.1	18	6	15	chromosome 20 open reading frame 112 (C20orf112)	X

NM_139016.2	1	9	9	chromosome 20 open reading frame 198 (C20orf198)	X
NM_144982.1	11	13	19	coiled-coil domain containing 131 (CCDC131)	X
BC064391.1	11	18	15	Coiled-coil domain-containing protein 6	X
NM_017949.1	28	9	13	CUE domain containing 1 (CUEDC1)	X
BC008668.1	41	3	3	cyclin G associated kinase (GAK)	X
NM_016508.2	26	13	17	Cyclin-dependent kinase-like 3	X
NM_022720.5	26	17	17	DiGeorge syndrome critical region gene 8 (DGCR8)	X
NM_014280.1	18	7	13	DnaJ homolog subfamily C member 8	X
NM_178151.1	19	16	13	doublecortex; lissencephaly, X-linked (doublecortin) (DCX), transcript variant 4	X
BC001105.1	6	13	7	dysbindin (dystrobrevin binding protein 1) domain containing 2 (DBNDD2)	X
NM_145245.1	5	15	7	ecotropic viral integration site 5-like (EVI5L)	X
NM_003792.1	19	15	5	endothelial differentiation-related factor 1 (EDF1), transcript variant alpha	X
BC006318.1	16	2	11	erythrocyte membrane protein band 4.9 (dematin) (EPB49)	X
BC052805.1	16	20	13	erythrocyte membrane protein band 4.9 (dematin) (EPB49)	X
NM_152789.1	17	5	5	family with sequence similarity 133, member B (FAM133B), transcript variant 1	X
BC070112.1	11	6	13	family with sequence similarity 53, member A (FAM53A)	X
NM_015122.1	9	9	7	FCH domain only 1 (FCHO1)	X
NM_021032.2	20	6	19	fibroblast growth factor 12 (FGF12), transcript variant 1	X
NM_054016.1	37	16	17	FUS interacting protein (serine/arginine-rich) 1 (FUSIP1), transcript variant 2	X
NM_007278.1	23	7	19	GABA(A) receptor-associated protein (GABARAP)	X
NM_031412.1	47	3	7	GABA(A) receptor-associated protein like 1 (GABARAPL1)	X
NM_005513.1	47	8	21	general transcription factor IIE, polypeptide 1, alpha 56kDa (GTF2E1)	X
BC000120.1	3	13	3	general transcription factor IIF, polypeptide 1, 74kDa (GTF2F1)	X
NM_022343.2	22	18	7	Golgi-associated plant pathogenesis-related protein 1	X
NM_198395.1	23	17	3	GTPase activating protein (SH3 domain) binding protein 1 (G3BP1), transcript variant 2	X
BC013959.1	19	13	1	guanine nucleotide binding protein-like 1 (GNL1)	X
NM_005526.1	24	18	21	heat shock transcription factor 1 (HSF1)	X
NM_016287.2	19	7	15	heterochromatin protein 1, binding protein 3 (HP1BP3)	X
NM_002136.1	23	3	17	heterogeneous nuclear ribonucleoprotein A1 (HNRNPA1), transcript variant 1	X
NM_144608.1	20	14	7	hexamthylene bis-acetamide inducible 2 (HEXIM2)	X
NM_145899.1	24	3	3	high mobility group AT-hook 1 (HMGA1), transcript variant 1	X
NM_138730.1	44	3	11	high mobility group nucleosomal binding domain 3 (HMGN3), transcript variant 2	X
BC011842.2	37	13	13	hypothetical protein FLJ11184 (FLJ11184)	X
NM_002266.2	26	17	19	Importin subunit alpha-2	X
NM_014790.3	3	5	9	janus kinase and microtubule interacting protein 2 (JAKMIP2)	X
BC094800.1	20	18	21	Jouberin	X
NM_018039.2	22	8	1	jumonji domain containing 2D (JMJD2D)	X
NM_018357.2	2	6	9	La ribonucleoprotein domain family, member 6 (LARP6), transcript variant 1	X
BC015586.2	40	8	11	laminin, gamma 1 (formerly LAMB2) (LAMC1)	X
NM_032563.1	31	6	17	late cornified envelope 3D (LCE3D)	X
NM_181714.1	20	9	15	Leber congenital amaurosis 5 (LCA5)	X
BC022983.1	27	4	21	ligand of numb-protein X 1 (LNX1)	X
NM_018032.2	47	17	5	LUC7-like (S. cerevisiae) (LUC7L), transcript variant 1	X

BC042625.1	18	4	21	LUC7-like 2 (S. cerevisiae) (LUC7L2)	X
NM_005565.2	16	14	15	lymphocyte cytosolic protein 2 (SH2 domain containing leukocyte protein of 76kDa) (LCP2)	X
NM_078630.1	33	7	1	male-specific lethal 3-like 1 (Drosophila) (MSL3L1), transcript variant 2	X
NM_002363.1	48	12	19	melanoma antigen family B, 1 (MAGEB1), transcript variant 1	X
NM_014061.3	27	15	1	melanoma antigen family H, 1 (MAGEH1)	X
NM_022474.2	24	7	19	membrane protein, palmitoylated 5 (MAGUK p55 subfamily member 5) (MPP5)	X
NM_004527.2	35	6	3	mesenchyme homeobox 1 (MEOX1), transcript variant 1	X
BC017423.1	11	17	13	mesoderm induction early response 1 homolog (Xenopus laevis) (MIER1)	X
BC026039.1	44	6	9	mitochondrial GTPase 1 homolog (S. cerevisiae) (MTG1)	X
BC009967.1	24	3	7	N-terminal kinase-like protein	X
U1snRNP68	18	15	13	NA	X
Centromere Protein B (CENP-B)	38	15	13	NA	X
PV3821	4	15	21	NIMA (never in mitosis gene a)-related kinase 3 (NEK3), transcript variant 1	X
BC006769.1	9	20	21	nucleolar and coiled-body phosphoprotein 1 (NOLC1)	X
NM_018454.4	20	20	11	nucleolar and spindle associated protein 1 (NUSAP1), transcript variant 2	X
NM_012387.1	4	18	17	peptidyl arginine deiminase, type IV (PADI4)	X
NM_001002913.1	27	2	11	peptidyl-tRNA hydrolase 1 homolog (S. cerevisiae) (PTRH1)	X
NM_002677.1	23	13	7	peripheral myelin protein 2 (PMP2)	X
NM_003688.1	34	10	9	Peripheral plasma membrane protein CASK	X
NM_004456.2	16	21	5	Polycomb protein EZH2	X
XM_378350.2	44	7	15	PREDICTED: Homo sapiens hypothetical gene supported by BC047417, transcript variant 1 (LOC400027)	X
XM_086879.4	28	8	5	PREDICTED: Homo sapiens hypothetical LOC150371 (LOC150371)	X
XM_379194.1	28	7	19	PREDICTED: Homo sapiens hypothetical LOC401068 (LOC401068)	X
NM_004397.3	18	21	19	Probable ATP-dependent RNA helicase DDX6	X
NM_018457.1	34	15	11	proline rich 13 (PRR13), transcript variant 2	X
NM_175887.2	40	6	7	proline rich 15 (PRR15)	X
NM_032636.2	40	12	17	proline/serine-rich coiled-coil 1 (PSRC1), transcript variant 1	X
NM_003720.1	47	8	5	Proteasome assembly chaperone 1	X
NM_173698.1	40	17	21	Protein FAM133A	X
BC035058.1	20	4	19	protein kinase, cAMP-dependent, catalytic, beta (PRKACB)	X
NM_199326.1	32	5	19	protein phosphatase 2 (formerly 2A), regulatory subunit B'', beta (PPP2R3B), transcript variant 2	X
BC027178.1	39	4	3	PRP40 pre-mRNA processing factor 40 homolog A (S. cerevisiae) (PRPF40A)	X
NM_003621.1	22	9	9	PTPRF interacting protein, binding protein 2 (liprin beta 2) (PPFIBP2)	X
BC103660.1	18	20	21	Putative RNA-binding protein 15	X
NM_002904.4	29	16	15	RD RNA binding protein (RDBP)	X
NM_004448.1	1	10	9	Receptor tyrosine-protein kinase erbB-2	X
BC094719.1	12	21	1	Rho GTPase-activating protein 12	X
BC010919.1	40	5	9	ribosomal protein L35 (RPL35)	X
NM_001022.3	22	9	13	ribosomal protein S19 (RPS19)	X
BC053365.1	38	3	21	ribosomal protein S6 kinase, 70kDa, polypeptide 1 (RPS6KB1)	X
NM_198467.1	43	7	3	round spermatid basic protein 1-like (RSBN1L)	X

BC000442.1	22	9	19	Serine/threonine-protein kinase 12	X
BC001280.1	5	2	15	Serine/threonine-protein kinase 6	X
NM_024800.1	22	9	21	Serine/threonine-protein kinase Nek11	X
BC002488.1	22	11	21	SERPINE1 mRNA binding protein 1 (SERBP1)	X
NM_030645.1	31	3	15	SH3-binding domain protein 5-like (SH3BP5L)	X
BC010947.1	47	17	17	signal recognition particle 19kDa (SRP19)	X
NM_001018116.1	27	8	5	similar to RIKEN cDNA 2310039E09 (LOC347273)	X
BC093990.1	47	18	13	Sin3 histone deacetylase corepressor complex component SDS3	X
BC031691.2	9	9	13	SLAIN motif family, member 2 (SLAIN2)	X
NM_003089.4	19	6	3	small nuclear ribonucleoprotein 70kDa polypeptide (RNP antigen) (SNRP70)	X
BC013778.1	33	4	19	solute carrier family 7, member 6 opposite strand (SLC7A6OS)	X
NM_006924.3	19	3	19	splicing factor, arginine/serine-rich 1 (splicing factor 2, alternate splicing factor) (SFRS1), transcript variant 1	X
BC000914.1	24	17	9	splicing factor, arginine/serine-rich 3 (SFRS3)	X
BC018823.2	33	3	5	splicing factor, arginine/serine-rich 5 (SFRS5)	X
NM_006275.2	41	12	15	splicing factor, arginine/serine-rich 6 (SFRS6)	X
BC000997.2	22	16	21	splicing factor, arginine/serine-rich 7, 35kDa (SFRS7)	X
NM_003769.2	14	8	1	splicing factor, arginine/serine-rich 9 (SFRS9)	X
NM_017503.2	33	13	15	surfeit 2 (SURF2)	X
NM_032943.2	20	7	11	synaptotagmin-like 2 (SYTL2), transcript variant a	X
NM_144659.1	14	3	15	t-complex 10 (mouse)-like (TCP10L)	X
NM_006398.2	22	18	9	Ubiquitin D	X
NM_006590.2	23	9	11	ubiquitin specific peptidase 39 (USP39)	X
NM_152653.1	24	3	19	ubiquitin-conjugating enzyme E2E 2 (UBC4/5 homolog, yeast) (UBE2E2)	X
NM_024099.2	2	6	7	Uncharacterized protein C11orf48	X
NM_138787.1	20	14	11	Uncharacterized protein C11orf74	X
NM_018553.1	15	8	7	Uncharacterized protein C17orf85	X
BC037311.1	41	17	5	Voltage-dependent L-type calcium channel subunit beta-1	X
NM_207519.1	11	8	13	zeta-chain (TCR) associated protein kinase 70kDa (ZAP70), transcript variant 2	X
BC034435.1	30	18	21	zinc finger CCCH-type containing 3 (ZC3H3)	X
BC026030.1	32	5	15	zinc finger protein 239 (ZNF239)	X
BC091489.1	28	18	19	zinc finger, MYND domain containing 11, mRNA (cDNA clone MGC:111056 IMAGE:6186814), complete cds	X
NM_203350.1	17	5	21	zinc finger, RAN-binding domain containing 2 (ZRANB2), transcript variant 1	X

Supplementary Methods

Reagents

Smoothened agonist SAG was purchased from Calbiochem. Trichostatin A (TSA), SANT (SANT-1) and Methylstat were from Sigma and JIB-04 from Axon Medchem. Latrunculin B, Phalloidin-CaliforniaRed was purchased from Biomol and N-Oxalyl-glycine (NOG), (+)-JQ1 were from Cayman Chemical.

Plasmids

GLI and DYRK1A expression plasmids and Hh reporter constructs have been described previously [7] {Mao, 2002 #42}. ABLIM plasmids were a kind gift of Dr. Norbert Frey (University Hospital Kiel, Germany). Plasmids encoding flag-tagged MKL1 (#11978) and NWASP (#33019) were obtained from Addgene. Cofilin (CFL1) and Profilin (PFN1) expression constructs were purchased from Thermo Scientific.

Cell lines

NIH3T3, Hek293T, Hek293A, ShhL2, C3H10T1/2, Panc1, A549, Daoy cell lines were purchased from ATCC. All cell lines were cultured in Dulbecco's Modified Eagle Medium (DMEM (high Glucose plus Glutamine and Pyruvate), Invitrogen) supplemented with 10 % fetal bovine serum (FBS) and 1 % Penicillin/Streptomycin at 37°C with 5 % CO_2. Immortalized wildtype and *Sufu-/-* MEFs were a kind gift of Rune Toftgard (Karolinska Institute, Stockholm, Sweden). $MEF^{[SHH]}$ cells were kindly provided by Wade Bushman [8].

All experiments were carried out in 10 % FBS containing media unless otherwise stated in the figure legend.

Co-immunoprecipitation experiments

Cells were transfected with plasmid and PEI (Sigma) and were lysed 48 h later in PBS/1% Triton X-100 containing protease inhibitor cocktail (Sigma P8340) (plus phosphatase inhibitors (Na-Molybdate (1mM), Na-Vanadate (1mM), NaF (20mM), β-Glycero-phosphate (25mM) (in those cases in which phosphorylation had to be preserved). Genomic DNA was sheared through a fine needle and cell debris were precipitated by centrifugation (10.000 g, 10 min, 4° C). The supernatant was precleared by addition of magnetic Dynabeads (Life

Technologies) and the corresponding normal IgG (Santa Cruz) for 1-3 h at 4° C. After magnetic removal of the beads, an aliquot was taken as input control and the rest was used for immunoprecipitation with the respective antibody plus Dynabeads (3 h, 4° C). Subsequently, beads were washed 4-6 times with lysis buffer, and boiled with SDS sample buffer for gel electrophoresis and immunoblotting.

Cloning, expression and purification of recombinant GLI1 N-terminal domain

Sequences of wildtype or mutant GLI1 N-terminal domain (aa 1-145) were PCR-amplified (Q5 high fidelity polymerase, NEB) and cloned into the His-tag-encoding bacterial expression plasmid pET-28a (Novagen). BL21(DE3) bacteria were transformed with the resulting plasmids and protein expression was induced for 4 h with 1 mM IPTG at 37 °C. Subsequently, bacterial pellets were lysed in B-PER (Thermo) plus protease inhibitors (Leupeptin (10 mg/ml); Aprotinin (10 mg/ml); PMSF (100 mM)) and purified under denaturing conditions using Protino Ni-IDA 150 columns according to the manufacturer (Macherey&Nagel).

In vitro kinase assay

0.25 µg Baculovirus-expressed, enzymatically active recombinant GST-tagged DYRK1A (Life Technologies, PV3785) was mixed with 1.5 µg bacterially expressed recombinant His-tagged GLI1 N-terminal domain plus 200 µM ATP (Sigma) in kinase buffer (25mM Tris (pH7.5), 10mM $MgCl_2$, 0.5mM EGTA, 0.5mM Na_3VO_4, 5mM ß-glycerophosphate, 2.5mm DTT, 0.01% Triton X-100) (30 µl total volume) and incubated at 30 °C for 20 min. Subsequently, samples were snap-frozen in dry-ice/Ethanol and sent for mass spectrometry.

Mass spectrometry

Protein samples were separated on denaturing gradient gels and bands from the Coomassie stained gel were excised manually, in gel digested, and extracted as described previously [9]. The extracted peptides were purified and concentrated using C18-Tips ™ columns (Supelco). The bound peptides were washed with 0.1 % trifluoroacetic acid, eluted from the column in 2 µl of 70% acetonitrile/ 0.1 % trifluoroacetic acid solution, and directly spotted onto a MALDI target plate prepared with α-cyano-4-hydroxycinnamic acid.

MS Analyses were performed using a Bruker Daltonics Ultraflex™ mass spectrometer equipped with a nitrogen laser (337 nm laser, 3-ns pulse width, and 50-Hz repetition rate) and panoramic mass range focusing (PAN™) technology and high precision calibration

(HPC™) for high mass accuracy. Peptide mass fingerprint spectra were acquired in the reflectron positive mode with a pulsed extraction using an average of 100 laser shots. The spectra were acquired after an external calibration using reference peptides (peptide mixture II, Bruker Daltonics). The spectra were further internally calibrated using trypsin autolysis peaks (842.5100 and 2211.1046 Da). Monoisotopic masses were assigned and processed using BioTools™ and flexAnalysis™ software before submission to the Mascot program (www.matrixscience.com) for searches against the Swiss-Prot Database. The following variable modifications were used in the searches: methionine oxidation, serine and threonine phosphorylation and a fixed cysteine carbamidomethylation modification. The searches were done allowing complete cleavage (0 missed cleavage sites) and allowing one or two missed cleavage sites. A mass accuracy of 100 ppm or better was used in all identifications.

Tandem mass spectrometry (MS/MS) analysis was done using the LIFT™ mode. The masses of the fragmented ions were submitted to the Mascot program for data base searching using the following parameters: peptide mass tolerance of 50 ppm, MS/MS tolerance of 0.7 Da, and one missed cleavage sites. The variable and fixed modifications of amino acid residues were used as described for the peptide mass fingerprint analyses.

Immunohistochemistry

Immunohistochemistry on formaldehyde-fixed and paraffin-embedded tissue sections was performed using antibodies listed in the supplemental section. Histological examination was done by a trained and certified pathologist (A.R.).

Supplementary References

1. Olson EN, Nordheim A. Linking actin dynamics and gene transcription to drive cellular motile functions. *Nat Rev Mol Cell Biol* **11**, 353-365 (2010).

2. Lundquist MR*, et al.* Redox modification of nuclear actin by MICAL-2 regulates SRF signaling. *Cell* **156**, 563-576 (2014).

3. Esnault C*, et al.* Rho-actin signaling to the MRTF coactivators dominates the immediate transcriptional response to serum in fibroblasts. *Genes Dev* **28**, 943-958 (2014).

4. Ho CY, Jaalouk DE, Vartiainen MK, Lammerding J. Lamin A/C and emerin regulate MKL1-SRF activity by modulating actin dynamics. *Nature* **497**, 507-511 (2013).

5. Medjkane S, Perez-Sanchez C, Gaggioli C, Sahai E, Treisman R. Myocardin-related transcription factors and SRF are required for cytoskeletal dynamics and experimental metastasis. *Nat Cell Biol* **11**, 257-268 (2009).

6. Oliver TG*, et al.* Loss of patched and disruption of granule cell development in a pre-neoplastic stage of medulloblastoma. *Development* **132**, 2425-2439 (2005).

7. Lauth M, Bergstrom A, Shimokawa T, Toftgard R. Inhibition of GLI-mediated transcription and tumor cell growth by small-molecule antagonists. *Proc Natl Acad Sci U S A* **104**, 8455-8460 (2007).

8. Lipinski RJ, Bijlsma MF, Gipp JJ, Podhaizer DJ, Bushman W. Establishment and characterization of immortalized Gli-null mouse embryonic fibroblast cell lines. *BMC Cell Biol* **9**, 49 (2008).

9. Hellman U, Wernstedt C, Gonez J, Heldin CH. Improvement of an "In-Gel" digestion procedure for the micropreparation of internal protein fragments for amino acid sequencing. *Analytical biochemistry* **224**, 451-455 (1995).
