# Supplementary material for: Identification of a novel actin-dependent signal transducing module allows for the targeted degradation of GLI1
Source: Nat Commun. 2015 Aug 27;6:8023. doi: 10.1038/ncomms9023 (PMC4552080; doi:10.1038/ncomms9023)
Supplement: Supplementary Data 1 — Primary antibodies and siRNA sequences [file ncomms9023-s2.docx]

**Supplementary Data 1**

Primary antibodies

| **Antibody** | **Dilution** | **Species** | **Clone/Epitope** | **Order#** | **Source** |
| --- | --- | --- | --- | --- | --- |
| **β-Actin** | 1:10.000 (WB) | mouse | AC-15 | A5441 | Sigma |
| **SUFU** | 1:1000 (WB) | rabbit | C81H7 | 2522 | Cell Signaling Technology |
| **Di/TriMeH3K9** | 1:100 (IF) | mouse | 6F12 | 5327 | Cell Signaling Technology |
| **HA-Tag** | 1:1000 (WB) | mouse | HA.11-16B12 | MMS-101P | Covance |
| **Myc-Tag** | 1:200 (IP) | mouse | 9E10 | sc-40 | Santa Cruz |
| **Myc-Tag** | 1:1000 (WB) | rabbit | 71D10 | 2278 | Cell Signaling Technology |
| **V5-Tag** | 1:5000 (WB) | mouse |  | R960-25 | Invitrogen |
| **V5-Tag** | 1:1000 (IP) | rabbit |  | A190-120A | Bethyl Laboratories |
| **Flag-Tag** | 1:1000 (WB) | mouse | M2 | F1804 | Sigma |
| **Flag-Tag** | 1:600 (IP) | rabbit |  | 600-401-383 | Rockland |
| **DYRK1A** | 1:1000 (WB) | rabbit |  | 2771 | Cell Signaling Technology |
| **(α)-Tub** | 1:1000 (WB) | mouse | DM1A | T6199 | Sigma |
| **MKL1** | 1:2000 (WB) | rabbit |  | A302-201A | Bethyl Laboratories |
| **Histone H2B** | 1:1000 (WB) | mouse | 53H3 | 2934 | Cell Signaling Technology |
| **HIP1** | 1:500 (WB) | goat |  | AF1568 | R&D Systems |
| **GFP** | 1:200 (IP)  1:1000 (WB) | mouse | B-2 | sc-9996 | Santa Cruz |
| **KDM3A** | 1:1000 (WB) | rabbit |  | PA5-23066 | Thermo Fisher Scientific |
| **GLI1** | 1:1000 (WB) | mouse | L42B10 | 2643 | Cell Signaling Technology |

ShRNA sequences (targeting human genes)

| **Name**  **(pLKO.1-backbone)** |  | | **Target sequence** | |
| --- | --- | --- | --- | --- |
| **shGFP (shCon)-Targeting GFP** | |  | | TACAACAGCCACAACGTCTAT |
| **shSUFU #2** | |  | | CCTTTCGTCTGAAGAGAGAAA |
| **shSUFU #5** | |  | | GACCGAAGAGTTTGTAGAGAA |
|  | |  | |  |

**siRNA sequences**

siRNAs: If not otherwise stated, equimolar pools of siRNAs #1-#4 were used.

SiRNA sequences (targeting mouse genes)

| **Name** |  | | **Target sequence** | |
| --- | --- | --- | --- | --- |
| **siCon (Targeting Firefly luciferase; siLuc)** | |  | | UAAGGCUAUGAAGAGAUAC |
| **siCon (Qiagen’s All-Star; siAll)** | |  | | AAUUCUCCGAACGUGUCACGU |
| **siCon (Sigma’s Universal neg. control#1; siUni)** | |  | | proprietary |
| **siCon (siMix)** | |  | | Equimolar mix of siAll, siLuc, siUni |
| **siDyrk1a_2** | |  | | GAAAUGAAGUACUACAUAG |
| **siDyrk1a_4** | |  | | GGAUGUAUCUUGGUUGAAA |
| **siAblim1_1** | |  | | CCACAGACUUCGCUCAGUA |
| **siAblim1_2** | |  | | CAACAGAGGGCGCAACAAA |
| **siAblim1_3** | |  | | GGAUCAACAUUUACCGAAA |
| **siAblim1_4** | |  | | GUGUAUAGCCGGCACAGUU |
| **siAblim2_1** | |  | | GGAAACUCCUCAAUGCAGA |
| **siAblim2_2** | |  | | GAUAACAUCUAUAGGAAAC |
| **siAblim2_3** | |  | | GUAACAAACCGAAUUCGUG |
| **siAblim2_4** | |  | | GAUGAUCGGUCCUACAAGC |
| **siMkl1_1** | |  | | GCAAUGGCAUGGUGAAGUU |
| **siMkl1_2** | |  | | GACCGAGGACUAUUUGAAA |
| **siMkl1_3** | |  | | UGAAGGAGGCUAUCAUUGU |
| **siMkl1_4** | |  | | CAGCACGGGUGAUGAGAAU |
| **siMkl2_1** | |  | | AGUCUGUGGCUCACGAAUU |
| **siMkl2_2** | |  | | GCACUUCGAGGAUAGAAAU |
| **siMkl2_3** | |  | | GUGUUUGGAUGGUUGACAA |
| **siMkl2_4** | |  | | ACUGGGAAGUUGAUAGCAA |
| **siSmo_1** | |  | | CAAUUGGCCUGGUGCUUAU |
| **siSmo_2** | |  | | GAGCGUAGCUUCCGGGACU |
| **siSmo_3** | |  | | GGAGUAGUCUGGUUCGUGG |
| **siSmo_4** | |  | | GCUACAAGAACUAUCGGUA |
| **siSrf_1** | |  | | GAGUAUUAGCUGACCCGAU |
| **siSrf_2** | |  | | UUGAGGAGAUGACGUGAAA |
| **siSrf_3** | |  | | GAUGAUCUGCUGACGUUUA |
| **siSrf_4** | |  | | GCACAGUGUUCCCGUCCGA |
| **siSmarca4_1** | |  | | GAGCGAAUGCGGAGGCUUA |
| **siSmarca4_2** | |  | | CAAACUGGGCGUAUGAAUU |
| **siSmarca4_3** | |  | | GAGACUAUCCUCAUUAUUC |
| **siSmarca4_4** | |  | | GAUCCUCACUGGCACAGAU |
| **siKdm2a_1** | |  | | GCACAAUGGACACGAUACU |
| **siKdm2a_2** | |  | | GGUAUGUGUUGGAGCGCUA |
| **siKdm2a_3** | |  | | GAAGAAAGGAUUCGGUACA |
| **siKdm2a_4** | |  | | GAACAAUCCCAGCGGCAAA |
| **siKdm3a_1** | |  | | GUAUGACAGUGAAGCGAUU |
| **siKdm3a_2** | |  | | GGAAGUAGACCUAGUCAAU |
| **siKdm3a_3** | |  | | CCUUGUUGGUUCAGAAGUA |
| **siKdm3a_4** | |  | | CGGUGUGGGUUUGGAGUAU |
| **siKdm4a_1** | |  | | GAACAUCCUACGACGAUAU |
| **siKdm4a_2** | |  | | GGACGUGGAAGCAGCGGAU |
| **siKdm4a_3** | |  | | GGAAGGACAGCACGGUUAU |
| **siKdm4a_4** | |  | | GUUCGUGAGUUCCGCAAGA |
| **siKdm5a_1** | |  | | GCAAAUGAGACAACGGAAA |
| **siKdm5a_2** | |  | | UGACAAUUGUGGAGCGCAU |
| **siKdm5a_3** | |  | | CAACACAUUUGGCGGAUUU |
| **siKdm5a_4** | |  | | AGAGGAAGGUGUUGGAUAU |
| **siNcoa3_1** | |  | | GGGAAGAACUGGAGCCGAU |
| **siNcoa3_2** | |  | | ACGAGGAGGUAUCGGGAGA |
| **siNcoa3_3** | |  | | CAGCCAAAGUCUUCACGUA |
| **siNcoa3_4** | |  | | GGACAGUCACCAUCGUUUA |
| **siLhx2_1** | |  | | GGGCCAAGUUCAGGCGCAA |
| **siLhx2_2** | |  | | CCUACAACCCUCACAGACU |
| **siLhx2_3** | |  | | CGUCAGAUGCCACGCUGCA |
| **siLhx2_4** | |  | | ACAAACGACUCUUACCAAC |
|  | |  | |  |

siRNA sequences (targeting human genes)

| **Name** |  | | **Target sequence** | |
| --- | --- | --- | --- | --- |
| **siDYRK1A_1** | |  | | UAAGGAUGCUUGAUUAUGA |
| **siDYRK1A_2** | |  | | GCUAAUACCUUGGACUUUG |
| **siMKL1_1** | |  | | AAACUGAGCUGAUUGAGCG |
| **siMKL1_2** | |  | | GAACUAUCCCAAAGUAGCA |
| **siMKL1_3** | |  | | GACAGAGGACUAUCUCAAA |
| **siMKL1_4** | |  | | GAUCGGAGCUGGUCAGGAU |
| **siGLI2_1** | |  | | GCACACCGCUGCUCAAAGA |
| **siGLI2_2** | |  | | GCACUGGCUUCUCUGACAA |
| **siGLI2_3** | |  | | UAACAUGCCUGUGCAGUGG |
| **siGLI2_4** | |  | | CCGCCUAGCAUCAGCGAGA |
|  | |  | |  |

qPCR primer sequences

| **Gene** | **Species** | **sequence (5’ 🡪 3’)** |
| --- | --- | --- |
| ***Rplp0 (P0)*** | murine | for: TGCACTCTCGCTTTCTGGAGGGTGT  rev: AATGCAGATGGATCAGCCAGGAAGG |
| ***Gapdh*** | murine | for:GGTGTGAACGGATTTGGCCGTATTG  rev: CCGTTGAATTTGCCGTGAGTGGAGT |
| ***Gli1*** | murine | for: CCCATAGGGTCTCGGGGTCTCAAAC  rev: GGAGGACCTGCGGCTGACTGTGTAA |
| ***Gli2*** | murine | for: TGAGGAGAGTGTGGAGGCCAGTAGCA  rev: CCGGGGCTGGACTGACAAAGC |
| ***Gli3*** | murine | ror: AAAGCGGGAAGAGTGCCTCCAGGT  rev: TGGCTGCTGCATGAAGACTGACCAC |
| ***Ptch1*** | murine | for: CGCCTTCGCTCTGGAGCAGATTTC  rev: TGAGGAGACCCACAACCAAAAACTTGC |
| ***Ptch2*** | murine | for:CCCGTGGTAATCCTCGTGGCCTCTAT  rev:TCCATCAGTCACAGGGGCAAAGGTC |
| ***Hip1*** | murine | for: TGGCTCCCATCGGCTCTTCATTCTA  rev: AGGCTTAGCAGGCCCCTTTCGTCTC |
| ***Wif1*** | murine | for: CACGAACCCAACAAGTGCCAGTGTC  rev: TTAAGTGAAGGCGTGTGTCGCTCCA |
| ***Ablim1*** | murine | for: GTGACCAACAGAGGGCGCAACAAAA  rev: TCATGTCGTTGCGTCTCCAAAGAGGT |
| ***Ablim2*** | murine | for: TGTGAAACTGCCCAAAGACGTGGAC  rev: AGGCGGTCAAACTCCTCGATGCTC |
| ***Mkl1*** | murine | for: ATAAGAGTGCTGATGGCCCTGGCTTG  rev: CTTCAGCAGAGATGTGGGGGTTGC |
| ***Mkl2*** | murine | for: GAGGAAGCCATCAAGCAGGCACGTA  rev: GAGGTCGTCCATCTGCTGACTGTGC |
| ***Smo*** | murine | for: GAGGAGCCATATTGCCCCAGGATGT  rev: TCCGGCCCAAACGCTTCTCTAACTC |
| ***Srf*** | murine | for: TCTCGTGACAGCAGCACAGACCTCA  rev: GAGGTGGGGGCATACATCACTGCAT |
| ***Smarca4*** | murine | for: AGCCGGTTGTGAGTGACGATGACAG  rev: CCTCGGGGTCAGGACTCAGGAATGT |
| ***Kdm2a*** | murine | \| for: TTTCACCTGCACTGGGCCAAGAA \| \| --- \| \| rev: CACGCAACGGACACCATGAGGA \| |
| ***Kdm3a*** | murine | for: CCTGAGAACCTGATGCCCACACAGA  rev: TTGGCCTTTATGCCCCATTTTGCTC |
| ***Kdm4a*** | murine | \| for: AACAGGCCCCTCACTGTGCTGTGT \| \| --- \| \| rev: CATTTCGGGGATCAGTGGCTTGG \| |
| ***Kdm5a*** | murine | \| for: TGCAGCCCAAAACTGCCAAAGG \| \| --- \| \| rev: TCAGCCATTTCTGCGGATACACCAA \| |
| ***Ncoa3*** | murine | \| for: TGAACAGCAGCGGTGGGCACTT \| \| --- \| \| rev: CCACGTCCTGATGATCCTGTGCAGT \| |
| ***Lhx2*** | murine | \| for: AATCCCGATGCCAAGGACTTGAAGC \| \| --- \| \| rev: GCCGTAAAAGGTTGCGCCTGAACTT \| |
| ***Acta2*** |  | for: CCTGGAGAAGAGCTACGAACTGCCTGA  rev: TTTCGTGGATGCCCGCTGACTC |
|  |  |  |
| ***Stringent species primers used for xenograft study:*** |  |  |
| ***Gli1*** | murine | \| for: GAGGATCCGGCAGGGAAGAG \| \| --- \| \| rev: TGCCGGCCATCTCCACGCCG \| |
| ***Gli2*** | murine | \| for: AAGTTGGGATGGGGCCCTG \| \| --- \| \| rev: AGGCTCATACTCTTTGGTGGC \| |
| ***Ptch1*** | murine | \| for: CCTCCTGGTCACACGAACAAT \| \| --- \| \| rev: GCTGTGCTTCGTATTGCCTG \| |
| ***Tbp*** | murine | \| for: CTTCCGCTGGCCCATAGT \| \| --- \| \| rev: ACGCCAAGAAACAGTGATGCT \| |
|  |  |  |
| ***Human primer sequences:*** |  |  |
|  |  |  |
| ***RPLP0 (P0)*** | human | for: CCTTCTCCTTTGGGCTGGTCATCCA  rev: CAGACACTGGCAACATTGCGGACAC |
| ***GLI1*** | human | for: TCTGGACATACCCCACCTCCCTCTG  rev: ACTGCAGCTCCCCCAATTTTTCTGG |
| ***PTCH1*** | human | for: CCGCCTTCGCTCTGGAGCAGATT  rev: TCTGAAACTTCGCTCTCAGCCACAGC |
| ***DYRK1A*** | human | for: GGAAAACGGGAGTACAAACCACCAGGA  rev: GACCGTATGACCTGACTCCCCAGCA |
| ***GLI2*** | human | \| for: tgg ccg ctt cag atg aca gat gtt g \| \| --- \| \| rev: cgt tag ccg aat gtc agc cgt gaa g \| |
| ***SUFU*** | human | \| for: ACATCCCCGAGCACTGGCACTACAT \| \| --- \| \| rev: CGAAAGGTCAACTCAAAGCCAAAACCA \| |
|  |  |  |
|  |  |  |
|  |  |  |
|  |  |  |
| ***Stringent species primers used for xenograft study:*** |  |  |
| ***GLI1*** | human | \| for: GAGGTCCCATCAGGGAGGAA \| \| --- \| \| rev: TGCCAGTCATTTCCACACCA \| |
| ***GLI2*** | human | \| for: TCAACCCTGTCGCCATTCAC \| \| --- \| \| rev: CCACATGAGCCGTGTCCAG \| |
| ***PTCH1*** | human | \| for: CCGGCCACACGCACAGC \| \| --- \| \| rev: TGGGCCTCGTAGTGCCGA \| |
| ***TBP*** | human | \| for: GTATCTGCTGGCGGTTTGG \| \| --- \| \| rev: GGCACTGCGGAGAAAATGA \| |
